# Downregulation of RORα by alcohol promotes TGFβ and α-SMA expression in mouse lung fibroblasts

**DOI:** 10.3389/fmed.2026.1719787

**Published:** 2026-02-04

**Authors:** Xian Fan, Hui Tao, Bum-Yong Kang, Nicolas Diaz, Kenkichi Baba, Gianluca Tosini, Justin Guo, Samantha M. Yeligar, Viranuj Sueblinvong

**Affiliations:** 1 Division of Pulmonary, Allergy, Critical Care, and Sleep Medicine, Department of Medicine, Emory University School of Medicine, Atlanta, GA, United States; 2 Division of Pulmonary, Allergy, Sleep, and Cystic Fibrosis, Department of Pediatrics, Emory University School of Medicine, Atlanta, GA, United States; 3 Department of Pharmacology and Toxicology, Morehouse School of Medicine, Atlanta, GA, United States; 4 Atlanta Veterans Affairs Health Care System, Decatur, GA, United States

**Keywords:** circadian signaling, ethanol, lung fibroblast, RORα, α-SMA

## Abstract

**Introduction:**

Chronic ethanol exposure increases susceptibility to fibroproliferative maladaptive repair following acute lung injury. Ethanol disrupts molecular circadian rhythms in multiple organs, contributing to liver steatosis and renal fibrosis. Because circadian disruption is linked to TGFβ activation and tissue fibrosis, we hypothesized that ethanol alters lung circadian signaling and promotes profibrotic responses in lung fibroblasts through modulation of TGFβ and α-SMA expression.

**Methods:**

Lung slices from PER2-luciferase reporter mice fed with 20% (v/v) ethanol in drinking water for 8 weeks or only water (control) for 8 weeks were analyzed for real-time bioluminescent PER2 rhythms over 7 days. Lungs from control and ethanol-fed C57BL/6J mice were collected every 4 h over 24 h to assess rhythmicity of selected core clock genes and selected profibrotic markers mRNA expression. Primary murine lung fibroblasts (PLF) were treated with ethanol and evaluated for circadian gene and protein expression. RORα function was interrogated using siRNA knockdown and pharmacological agonist/inverse agonist, followed by analysis of TGFβ, α-SMA, and fibronectin protein levels.

**Results:**

Chronic ethanol ingestion lengthened the circadian period by ~2 h (*p* < 0.05) and induced a ~7% phase shift in PER2 rhythms in lung slices. Ethanol altered oscillatory patterns of core clock genes (*Bmal1, Clock, Rorα, Rev-erbα*) and profibrotic markers (*Tgfβ, α-SMA, Fn1*) in mouse lungs. *In vitro*, ethanol suppressed BMAL1 and RORα expression in PLF. Activation of RORα with agonist SR1078 reversed ethanol-induced TGFβ and α-SMA upregulation, whereas RORα reverse agonist (SR3335) mimicked ethanol’s effects. Lastly, the silencing of RORα gene expression significantly induced TGFβ and α-SMA, with a trend toward an increase in Fn1.

**Conclusion:**

Ethanol disrupts circadian signaling and enhances profibrotic gene expression in lung fibroblasts, partly through suppression of RORα. RORα activation mitigates these effects, identifying RORα as a potential therapeutic target for ethanol-related maladaptive lung repair.

## Introduction

The circadian signaling pathway plays a pivotal role in regulating various physiological processes in the human body, including sleep–wake cycles, hormone secretion, and cellular repair mechanisms (1). This intrinsic time-keeping system is governed by a set of core clock genes that generate rhythmic oscillations over a 24-h period (2). In the lung, the circadian clock has been implicated in modulating immune responses and tissue repair, particularly following acute lung injury (ALI) (3). Dysregulation of circadian signaling can lead to impaired lung repair mechanisms, exacerbating inflammatory responses, and contributing to the development of chronic lung diseases (4–6). Studies have shown that disruptions in circadian signaling can alter the timing of cell proliferation, cytokine release, and the activation of repair pathways, all of which are crucial for effective lung recovery post-injury (6).

The circadian signaling pathway is governed by two core transcription factors, BMAL1 and CLOCK, which regulate rhythmic expression of downstream clock genes such as PER, CRY, ROR, and REV-ERB, initiating a cascade of transcriptional events (7). Emerging evidence suggests a complex interplay between circadian rhythms and ethanol exposure, indicating that ethanol may influence tissue injury and repair by disrupting molecular clocks (8). Ethanol impairs circadian regulation by altering clock gene expression and disturbing both central and peripheral clock function (9). This misalignment promotes oxidative stress, inflammation, and immune dysregulation, compromising lung tissue integrity and repair (8). Chronic ethanol consumption is associated with increased prevalence and mortality from acute respiratory distress syndrome (ARDS) (10). Although direct evidence linking alcohol consumption to pulmonary fibrosis is lacking, our rodent model demonstrates that chronic ethanol ingestion leads to fibroproliferative maladaptive repair following acute lung injury. We further show that ethanol exposure, *in vivo* and *in vitro*, upregulates transforming growth factor-β (TGFβ) in epithelial cells and lung fibroblasts, promoting fibroblast-to-myofibroblast differentiation (11–13). Ethanol also disrupts epithelial barrier integrity and extracellular matrix homeostasis, resulting in persistent collagen deposition and impaired resolution of inflammation (14–16). Mechanistically, ethanol-induced oxidative stress and altered immune signaling, including dysregulated innate lymphoid cell activity, create an environment favoring pathological tissue remodeling and fibrosis (13, 17, 18). Despite these observations, no studies have directly examined how ethanol-induced circadian disruption in the lung contributes to injury and impaired repair.

The retinoid acid-related orphan receptor (ROR) family consists of three members of the nuclear receptors; RORα, β, and γ, that function as transcription factors and play important roles in maintaining circadian rhythm, regulating oxidative stress responses, and supporting tissue repair (19). While RORβ is primarily confined to the central nervous system and RORγ to immune cells, RORα is widely expressed across multiple tissues, including the lung, and plays a central role in regulating circadian gene expression. It also influences lipid metabolism, inflammation, and oxidative stress in peripheral tissues (7, 20). ROR and REV-ERB compete for binding to the ROR response element (RORE) to regulate BMAL1 expression, thereby stabilizing the core circadian clock and downstream physiological functions (21). Previous studies have demonstrated that RORα is critical for various physiological processes, including cell differentiation and inflammatory regulation (1). For example, overexpression of RORα suppresses epithelial-to-mesenchymal transition in a breast cancer model (21, 22). However, little is known about the expression and function of ROR family members in ethanol-induced lung diseases.

In this study, we examined how ethanol-induced circadian disruption dysregulates RORα, promoting lung fibroblast’s profibrotic phenotype with upregulation of TGFβ and α-SMA expression. We speculated that disruption of the circadian signaling pathway may underlie the exaggerated fibroproliferative response following acute lung injury in ethanol-exposed experimental model and that targeting circadian signaling could provide therapeutic strategies to improve lung repair.

## Materials and methods

### Animals and chronic ethanol ingestion

Two-month-old male and female period 2 (PER2) luciferase mice (PER2: Luc; PER2 protein fused with firefly luciferase) and C57BL/6J mice were from Jackson Laboratories (Bar Harbor, ME). A total of 16 male and six female PER2: Luc mice were randomly divided into two groups: water-fed as control-fed and 20% (v/v) ethanol in drinking water as ethanol-fed in a 12-h light–dark cycle. After 8 weeks of ethanol administration, lungs were isolated for bioluminescence measurement. In parallel, 88 male and female C57BL6/J mice were randomly divided into two groups for circadian oscillation study: control-fed and ethanol-fed for 8 weeks as described previously. At the end of the experiment, mouse lungs were collected every 4 h interval over 24 h for both control and ethanol fed mouse groups. All studies were approved by the Institutional Animal Care and Use Committee (IACUC) at Emory University, Atlanta, GA, United States of America (United States), and adhered to the Association for Assessment and Accreditation of Laboratory Animal Care International (AAALAC) standards for the humane treatment of laboratory animals. All animal experiments were conducted in compliance with the Animal Research: Reporting of *In Vivo* Experiments (ARRIVE) guidelines to ensure transparency, reproducibility, and ethical standards.

### Cell culture and treatment

Mouse primary lung fibroblasts (PLFs) were isolated from the lungs of three-month old C57BL/6J mice (23). Cells were cultured and expanded in DMEM with 20% FBS, 100 U/mL penicillin, and 100 U/mL streptomycin (24). Primary lung fibroblasts (PLFs) were used between passages 3 and 8, based on publications indicating that normal lung fibroblasts do not exhibit senescence markers until approximately passages 12–15 (23, 25, 26). Most cell culture experiments were under synchronized conditions. In some experiments, unsynchronized cells (indicated in result section and figures) were treated with or without ethanol and compared to synchronized cells, which were subjected to serum shock using DMEM containing 50% FBS for 2 h prior to treatment. Following synchronization, cells were washed and returned to normal culture media as previously described (27). Subsequent studies were performed in a synchronized condition. Cells were treated with DMSO or 5 μM of RORα agonist (SR1078, TOCRIS bioscience/Bio-Techne, Minneapolis, MN) or RORα inverse agonist (SR3335, Cayman Chemical, Ann Arbor, MI) in DMEM with 5% FBS for 30 min and then subjected to ethanol at 60 mM (23) in closed alcohol container in tissue culture incubator. The 60 mM ethanol concentration was selected based on it is physiologically relevant to humans, corresponding to a blood ethanol level of approximately 0.24–0.27%, which is typical for an inebriated adult. Furthermore, previous dose-titration studies demonstrated that 60 mM is the optimal concentration for inducing fibronectin activation in lung fibroblasts (23, 28, 29).

### Real-time bioluminescent measurement

Lungs were dissected from Per2: Luc mice fed ± ethanol and sliced approximately 3 mm using No. 11 Scalpels, placed on the culture membranes (Millicell-CM) in 35 mm petri dishes containing 1.2 mL of DMEM with 10 mm HEPES (pH 7.2), 0.1 mM D-Luciferin K salt, penicillin/streptomycin cocktail (2.5 mL/L), and B27 (2%), sealed, and maintained at 37 °C. Bioluminescence was measured for 1 min at 10-min intervals with a microplate luminometer equipped with photomultiplier tubes (Lumicycle^®^). Data were detrended using a 24 h moving average-subtraction method and smoothed with a 2-h moving average. Daily peaks were identified by Origin® software. The first peak observed in the smoothed data after 24 h *in vitro* was used as a phase marker (30). Circadian periods were determined from the slope of a linear regression line fitted to five consecutive circadian peaks.

### Silencing RORα

Mouse PLFs were transfected with 10 nM of either Stealth RNAi siRNA targeting mouse RORα or Stealth RNAi negative control, Med GC (Invitrogen, Carlsbad, CA) using Lipofectamine 3000 (Invitrogen) in Opti-MEM as described by manufacturer. After 6 h of transfection, DMEM supplemented with 10% FBS was added (final FBS concentration: 5%) ± 60 mM ethanol for 72 h for protein analysis. We tested the dose and time of three Stealth RNAi siRNA to mouse *Rorα*. All three *Rorα* siRNA significantly suppressed the mouse *Rorα* expression (data no showed).

### Total RNA isolation and expression analysis

Total RNA was isolated from mouse lungs and PLFs using Quick-RNA MicroPrep kit (Zymo Research, Irvine CA) (31). First-strand cDNA was synthesized with iScript cDNA Synthesis kit (Bio-Rad Laboratories, Hercules, CA), and real-time PCR was for *18s*, *Bmal1*, *Clock*, *Cryp1/2*, *Per1/2*, *Rorα*, *Rev-erbα/β, tgfβ, α-sma, and fn1* using Bio-Rad iQ SYBR Green SuperMix (murine qPCR primers in Table 1). Target mRNA expression was normalized to housekeeping gene, *18s* and relative expression were calculated using Delta–Delta Ct analysis (2^-ΔΔCt^) (24). Rhythmicity in gene expression was analyzed using the JTK_CYCLE algorithm implemented in the Nitecap platform^1^ as previously described (32). This algorithm detects rhythmic oscillations by comparing temporal expression patterns to reference cosine waveforms. The expression pattern with a JTK_CYCLE *p*-value < 0.05 were considered to exhibit statistically significant circadian rhythmicity.

**Table 1:** Murine primers for qPCR.

Name	Oligo sequence
*18s*	Forward: 5′-GGA CCA GAG CGA AAG CA − 3′ Reverse: 5′-ACC CAC GGA ATC GAG AAA − 3′
*Rorα*	Forward: 5′-CGC AGC GAT GAA AGC TCA AA-3′ Reverse: 5′-CAT CTC GAG ACA TCC CCA CG-3′
*Bmal1*	Forward: 5′-CCG TGC TAA GGA TGG CTG TT-3′ Reverse: 5′-CCT CGG TCA CAT CCC TGA GA-3′
*Clock*	Forward: 5′-TGG TGT TTA CCG TAA GCT GT-3′ Reverse: 5′-GAA GCA TAG ACC CCA GCT CC-3′
*Rev-erbα*	Forward: 5′-ACA AGC AAC ATT ACC AAG CTG A-3′ Reverse: 5′-CAC TCC ATA GTG GAA GCC TGA-3′
*Rev-erbβ*	Forward: 5′-CAA CGG CAA TCC CAA GAA CG-3′ Reverse: 5′-TGC TCC TCC GAA AGA AAC CC-3′
*Per1*	Forward: 5′-CGT TGC AAA CGG GAT GTG TT-3′ Reverse: 5′-GAC CTC CTC TGA TTC GGC AG-3′
*Per2*	Forward: 5′-AGA CGT GGA CAT GAG CAG TG-3′ Reverse: 5′-GAA GGC ATC ATC AGG GCT GG-3′
*Cry1*	Forward: 5′-CCG CTG CGT CTA TAT CCT CG-3′ Reverse: 5′-TGA AGC AAA AAT CGC CAC CTG-3′
*Cry2*	Forward: 5′-TCC TGA GAC TGG AGA GCC AT-3′ Reverse: 5′-GCT CCG TCT GTT GGT GAT TG-3′
*Tgfβ*	Forward: 5′-CCG TGG CTT CTA GTG CTG AC-3′ Reverse: 5′-GAC TGG CGA GCC TTA GTT TG-3′
*α-sma*	Forward: 5′-CTG ACA GAG GCA CCA CTG AA-3′ Reverse: 5′-CAT CTC CAG AGT CCA GCA CA-3′
*fn1*	Forward: 5′-AAT GGA AAA GGG GAA TGG AC-3′ Reverse: 5′-CTC GGT TGT CCT TCT TGC TC-3′

### Protein isolation and Western blot analyses

Total protein was isolated using Laemmli sample buffer as previously described (11). Proteins were separated on precast 4–20% SDS-PAGE gel (Bio-Rad), transferred to PVDF membranes. The membranes were blocked with 5% milk/TBS with 0.1% Tween 20 then incubated with antibodies for α-SMA (ab5694 at 1:3000, Abcam Waltham, MA), TGFβ (555,052 at 1:500, BD Pharmingen), fibronectin (sc-9068 at 1:1000, Santa Cruz Biotechnology), BMAL1 (MA5-25133 at 1:500, Invitrogen), CLOCK (5,157 at 1:1000, Cell Signaling), RORα (82930-1-RR at 1:1000, Proteintech), REV-ERBα (14506-1-AP at 1:1000, Proteintech), Per1 (sc-398890 at 1:500, Santa Cruz Biotechnology), Per2 (PA5-100107 at 1:500, Invitrogen), or GAPDH (G9545 at 1:50,000, Sigma-Aldrich) respectively, then incubated with an appropriate secondary antibody and exposed to Clarity Western ECL substrate (Bio-Rad Laboratories). The immunoreactive bands were captured using a ChemiDoc XRS system (Bio-Rad Laboratories) and analyzed relative densitometry, normalized to the GAPDH signal from the same membrane.

### Statistical analysis

All data are presented as means ± standard error (SE). For comparisons between two groups, the unpaired *t*-test with Welch’s correction was used when data met parametric assumptions; otherwise, the Mann–Whitney *U* test was applied. For multiple group comparisons, Kruskal–Wallis one-way ANOVA was used for non-parametric data, followed by Dunn’s multiple comparisons test. Statistical significance was defined as *p* < 0.05. Statistical analyses were carried out using GraphPad Prism, Version 7.0 software (La Jolla, CA).

## Results

### Chronic ethanol ingestion disrupts circadian oscillations in mouse lungs

**Figure 1:**
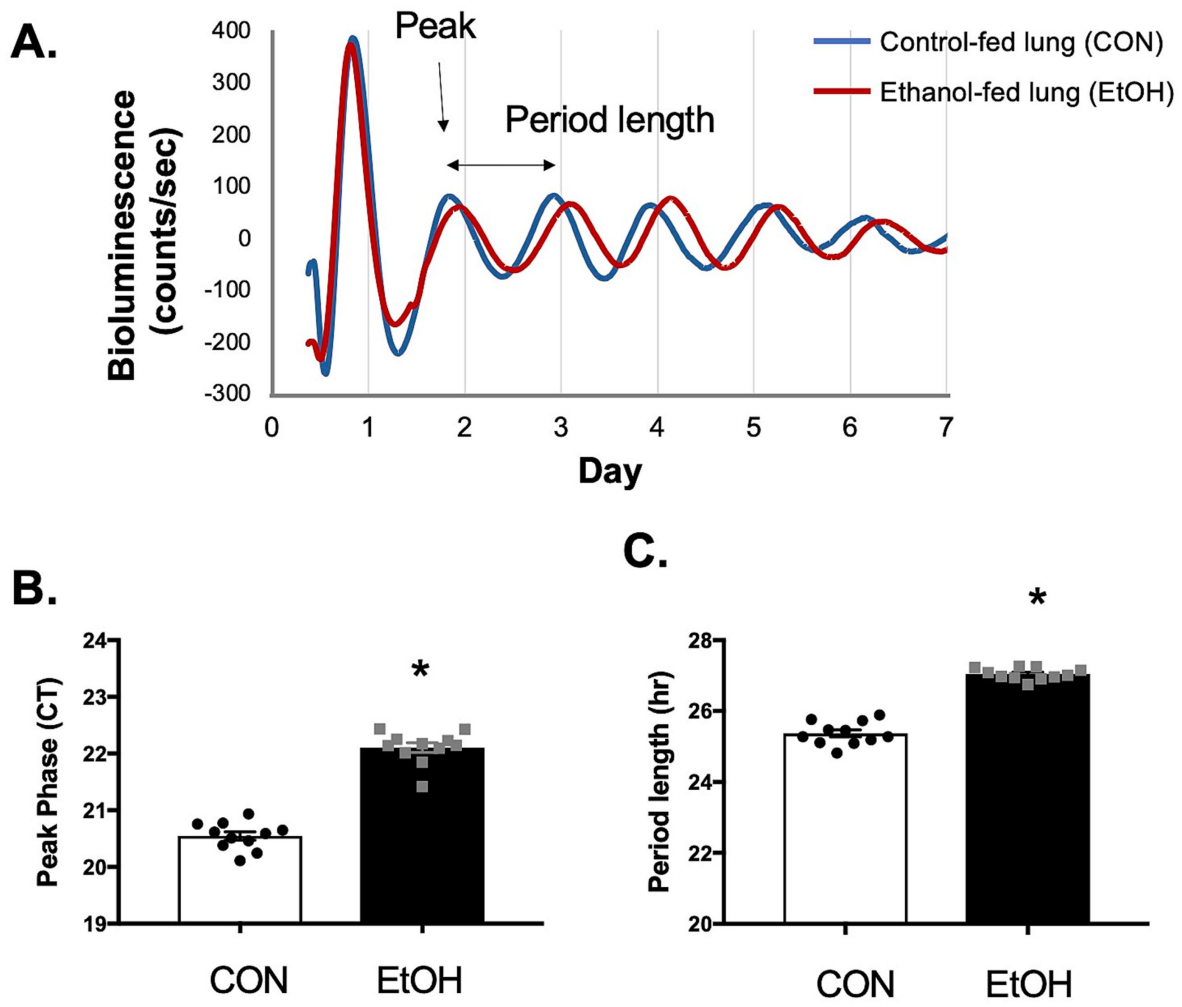
Chronic ethanol ingestion disrupted circadian rhythms in the murine lung. Per2: LUC bioluminescence from cultured lung slices of Per2: LUC mice were recorded using a Lumicycle for 7 days. **(A)** Representative Per2: LUC rhythms from ethanol-fed mice (20% v/v EtOH, red) and control-fed mice (CON; water, blue). Graphs summarize data for **(B)** peak phase and **(C)** circadian period (hours) of Per2: LUC rhythms in control-fed (CON, open bars) and ethanol-fed (EtOH, closed bars) groups. Both peak phase and circadian period significantly increased. *N* = 11 lung slices per group (one slice per mouse). Data are presented as mean ± SE. * Indicates change with *p* < 0.05 compared to control-fed group (CON).

To evaluate the impact of chronic ethanol consumption on pulmonary circadian rhythms, we measured real-time bioluminescence in lung slices from PER2: LUC reporter mice fed ethanol-containing drinking water or control water for 8 weeks. An initial pilot study included six males and six females divided into two groups; data were analyzed separately and revealed no significant sex-based differences (data no showed). A subsequent study was conducted using male mice. Because no sex differences were observed in bioluminescence patterns, the data were combined from two separate experiments. Both control and ethanol-fed groups exhibited robust PER2: LUC rhythms (Figure 1A); however, ethanol-fed mice demonstrated a significant delay in peak phase (22.2 ± 0.27 counts/s) compared to controls (20.5 ± 0.23 counts/s) (Figure 1B), along with an approximately 2-h lengthening of the circadian period (Figure 1C).

### Chronic ethanol ingestion alters circadian oscillation patterns of profibrotic gene expression in the lungs

**Figure 2:**
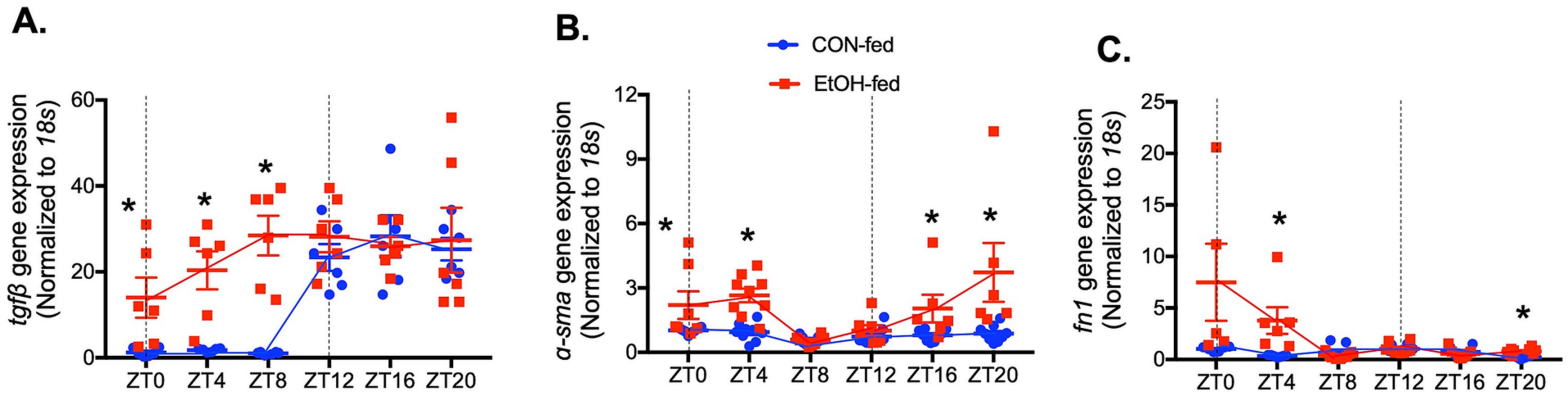
Chronic ethanol ingestion modified the oscillation pattern of profibrotic gene expressions in the mouse lungs. Graphs summarize gene expression profiles of profibrotic markers in mouse lungs at 4-h intervals over 24 h for ethanol-fed (EtOH, red) and control-fed (CON, blue) groups. Panels show expressions of **(A)** *Tgfβ*, **(B)** *α-SMA*, **(C)** *fn1* analyzed by qPCR. *N* = 6–9 lungs per group. All three genes from ethanol-fed mouse lungs significantly changed oscillation patterns compared to lungs from control-fed mice. Data are presented as mean ± SE. * Indicates change with *p* < 0.05 compared to control-fed (CON) group at the same time point.

To determine whether ethanol-induced circadian rhythm disruption affects *Tgfβ*, a profibrotic growth factor commonly elevated in fibrotic diseases, we examined the oscillatory patterns of profibrotic markers in lungs collected every 4 h over a 24-h period. In control-fed mice, *Tgfβ* expression exhibited a clear diurnal rhythm, with higher levels during the active phase (dark hours) and lower levels during the inactive phase (light hours) (Figure 2A). Although ethanol-fed mice retained some diurnal variation, *Tgfβ* levels were significantly elevated compared to controls during the inactive phase (light hours) (Figure 2A). Parallel analyses of *α-sma*, and fibronectin (*Fn1*) revealed altered oscillatory patterns in lungs of ethanol-fed mice relative to controls (Figures 2B–C). Specifically, ethanol inhibited *Tgfβ* rhythmicity while inducing the rhythmicity of *α-sma* and *Fn1* (Supplementary Table S1).

**Figure 3:**
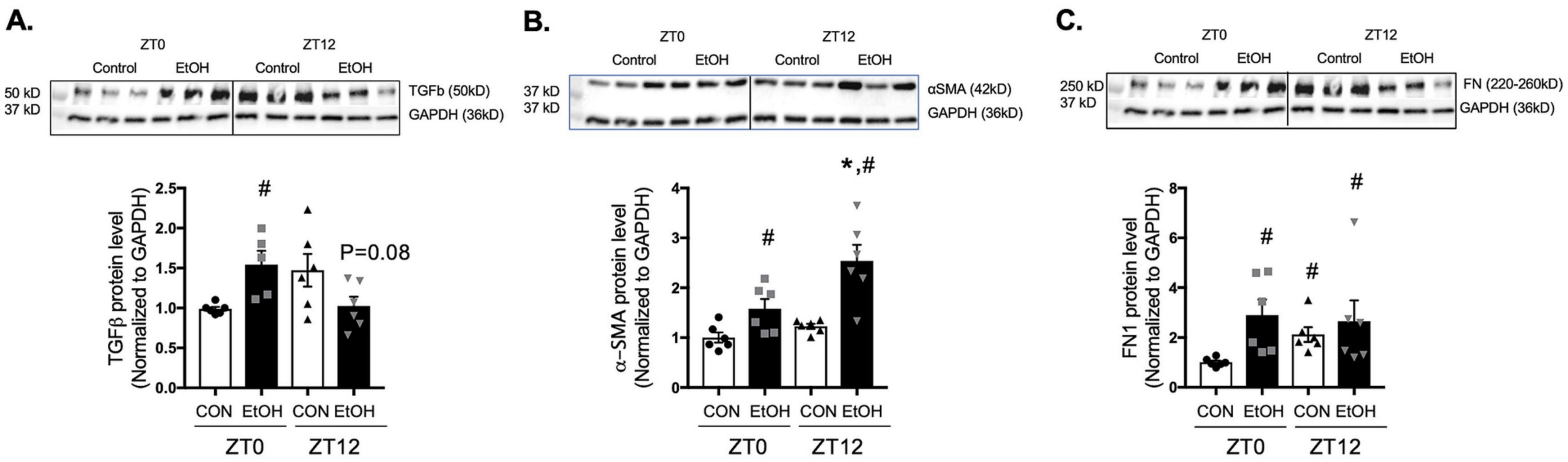
Chronic ethanol ingestion disrupts temporal protein expression of profibrotic molecules in the mouse lungs. Protein levels from mouse lungs collected at ZT0 and ZT12 were analyzed for **(A)** TGFβ (55 kD), **(B)** α-SMA (42 kD), and **(C)** fibronectin (220–260 kD) and normalized with GAPDH (36 kD) levels in the same blot by western analysis. All data reported as fold-change compared to control-fed at ZT0 (CON) group. Representative western immunoblots are shown above the corresponding graphs. *N* = 6 lungs per group. Data are presented as mean ± SE. ^#^ indicates change with *p* < 0.005 compared with ZT0 CON group and * indicates changes with *p* < 0.05 compared with ZT12 CON group.

In parallel, protein expressions were assessed at ZT0 and ZT12 to determine whether ethanol-induced changes in transcription were reflected at the protein level. Consistent with the early-phase increase in gene expression, TGFβ protein was significantly upregulated by ethanol at ZT0, whereas no difference was observed between control-fed and ethanol-fed lungs at ZT12 (Figure 3A). Ethanol similarly increased both α-SMA and fibronectin protein abundance at ZT0 compared with control lungs (Figures 3B,C). By ZT12, however, only α-SMA remained elevated in ethanol-fed lungs compared to the levels of control-fed lungs at ZT0 and ZT12, while fibronectin levels were comparably increased in both control-fed and ethanol-fed groups at ZT12, indicating a time-dependent divergence in protein regulation (Figures 3B,C). These observations suggest that chronic ethanol ingestion may alter the temporal regulation of profibrotic genes, especially TGFβ, α-SMA, and fibronectin, potentially contributing to fibroblast-to-myofibroblast differentiation (FMD).

### Chronic ethanol ingestion disrupts the circadian oscillation patterns of key clock genes in mouse lungs

**Figure 4:**
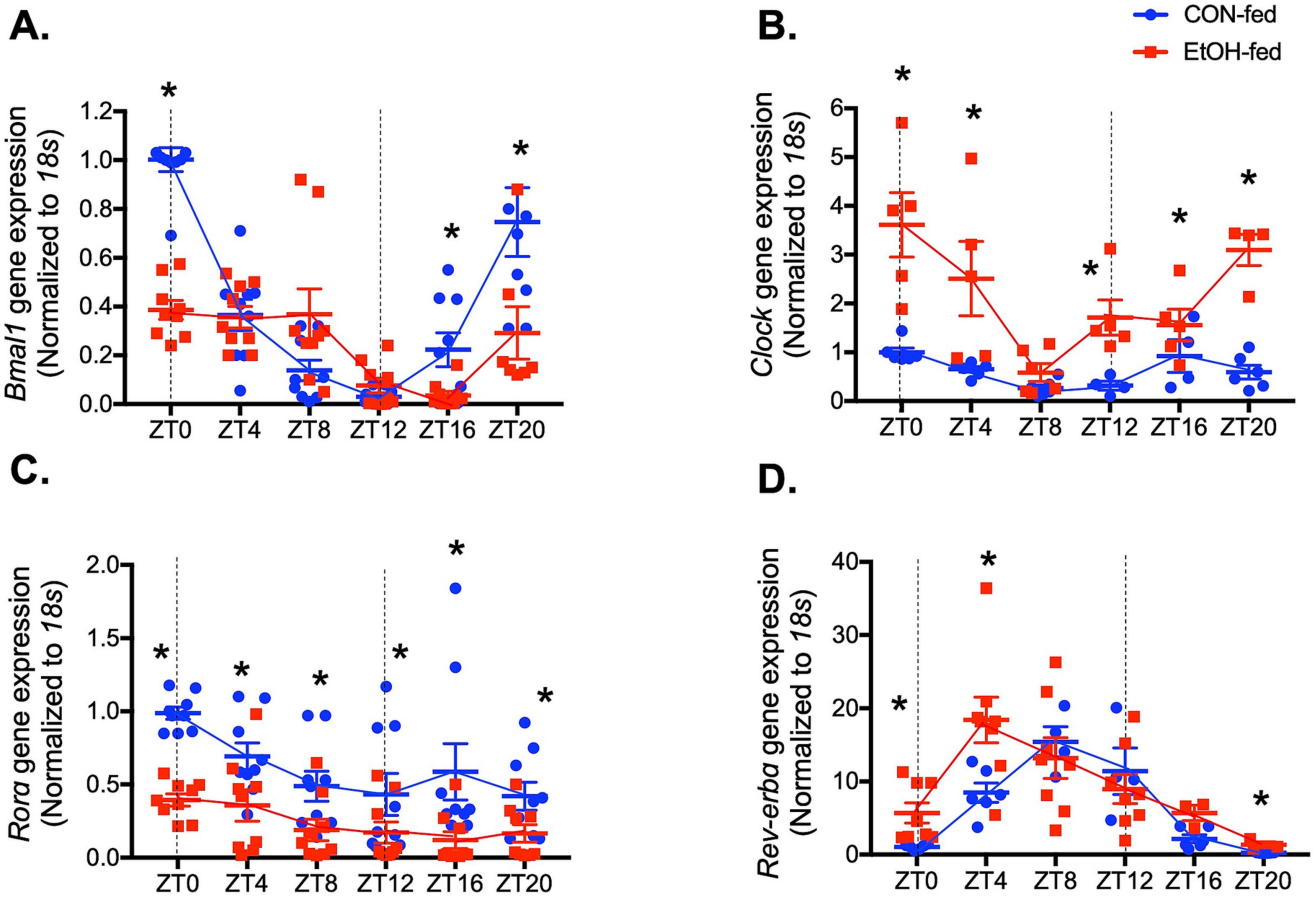
Chronic ethanol ingestion altered the oscillation patterns of key circadian signaling molecules in mouse lungs. Graphs summarize expression profiles of key circadian regulators in mouse lungs at 4 h intervals over 24 h for ethanol-fed (EtOH, red) and control-fed (CON, blue) groups. Panels show expressions of **(A)** *Bmal1*, **(B)** *Clock*, **(C)** *Rorα*, and **(D)** *Rev-erbα* analyzed by qPCR. *N* = 6–9 lungs per group. The circadian oscillation patterns of the four genes from ethanol-fed mouse lungs significantly changed compared to lungs from control-fed mice. Data are presented as mean ± SE. * Indicates change with *p* < 0.05 compared to control-fed (CON) group at the same time point.

To determine whether ethanol-induced alteration in the oscillatory patterns and increases of *Tgfβ* and *α-sma* are associated with altered circadian gene expression, we analyzed the same lung tissues collected every 4 h over a 24-h period from control and ethanol-fed mice. As shown in Figures 4A–D, the expression patterns of *Bmal1*, *Clock*, *Rorα*, and *Rev-erbα* were significantly altered in ethanol-fed mouse lungs compared to controls. Specifically, *Bmal1* expression was reduced from midnight to early morning (Figure 4A), while *Rorα*, a positive regulator of *Bmal1*, remained consistently low across all time points (Figure 4C). In contrast, *Rev-erbα* (*Bmal1 suppressor*) (Figure 4D) and *Clock* (Figure 4B) were significantly upregulated during the same time window.

**Figure 5:**
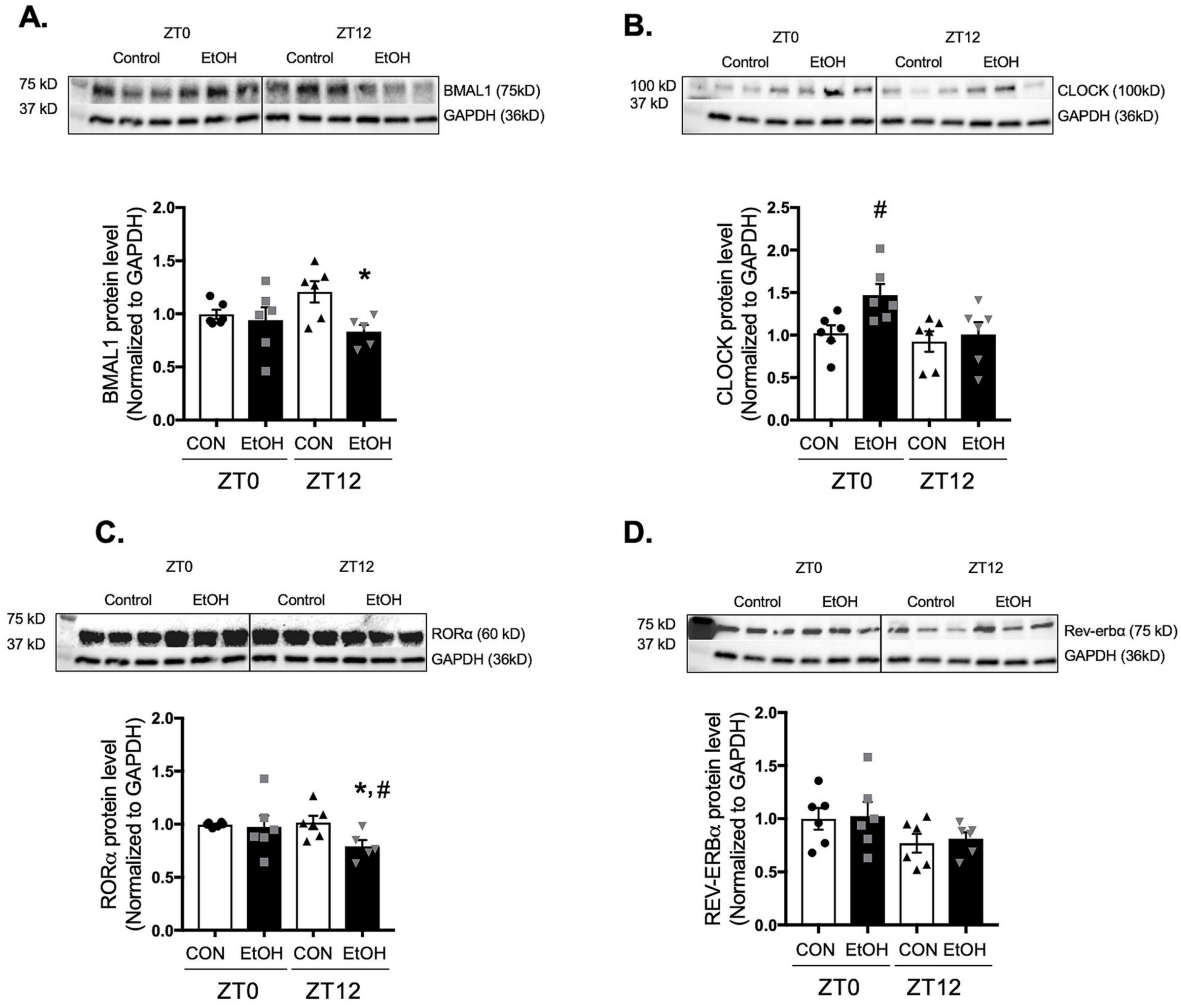
Chronic ethanol ingestion disrupts temporal protein expression of key circadian signaling molecules in the mouse lungs. Protein levels from mouse lungs collected at ZT0 and ZT12 were analyzed for **(A)** BMAL1 (75 kD), **(B)** CLOCK (100 kD), **(C)** RORα (60 kD), and **(D)** REV-ERBα (75 kD) and normalized with GAPDH (36 kD) levels in the same blot by western analysis. All data reported as fold-change compared to control-fed at ZT0 (CON) group. Representative western immunoblots are shown above the corresponding graphs. *N* = 6 per group. Data are presented as mean ± SE. ^#^ indicates change with *p* < 0.005 compared with ZT0 CON group and * indicates changes with *p* < 0.05 compared with ZT12 CON group.

In parallel, protein expression was assessed at ZT0 and ZT12 to determine whether ethanol-induced changes in core clock gene transcription were reflected at the protein level. Consistent with the phase-shifted mRNA suppression observed across several time points, BMAL1 protein was significantly reduced at ZT12 in ethanol-fed lungs compared with controls (Figure 5A). CLOCK protein, despite broad transcriptional upregulation across the cycle, was elevated only at ZT0 (Figure 5B), indicating restricted or time-specific translation. RORα protein was suppressed at ZT12 (Figure 5C), aligning only partially with its uniformly reduced mRNA levels, while REV-ERBα protein remained unchanged at both ZT0 and ZT12 despite rhythmic increases in its transcript (Figure 5D). Together, these findings demonstrate that chronic ethanol ingestion disrupts the normal coordination between circadian gene transcription and protein abundance, suggesting a broader dysregulation of the molecular clock that may underline the altered rhythmicity of profibrotic markers in the lung.

### Ethanol exposure suppresses key molecules of the circadian signaling pathway in lung fibroblasts

**Figure 6:**
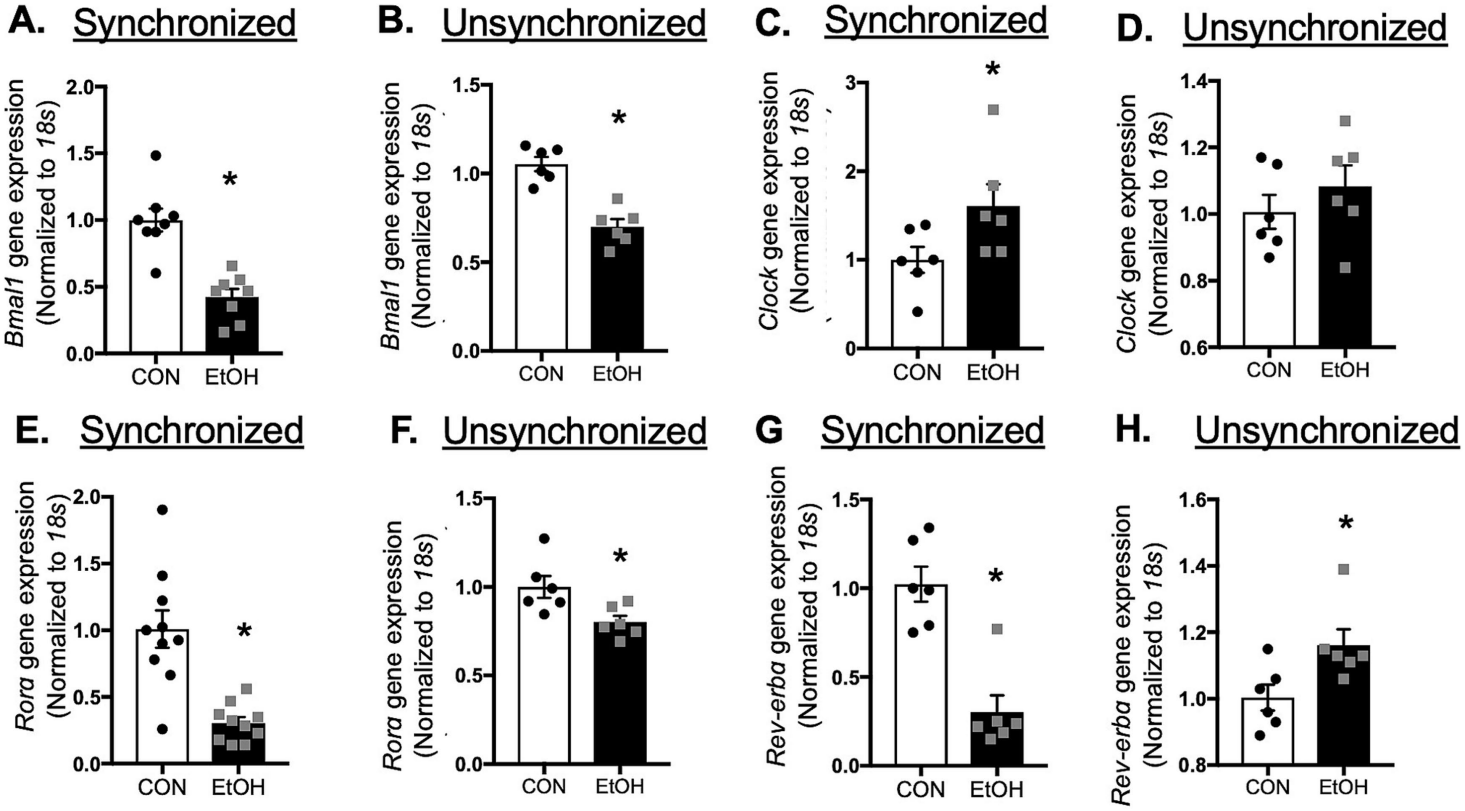
Ethanol exposure *in vitro* inhibited key molecules in the circadian signaling pathway. Gene expression levels from synchronized and unsynchronized murine primary lung fibroblasts (mPLFs) ± ethanol (EtOH, 60 mM; 24 h) were analyzed for: **(A,B)** *Bmal1*, **(C,D)** *Clock*, **(E,F)** *Rorα*, and **(G,H)** *Rev-erbα* mRNA expression. Ethanol exposure significantly inhibited *BMAL1, RORα,* and *Rev-erbα* gene expressions while upregulating *Clock* gene expression. *N* = 6–10 per group. Data are presented as mean ± SE. * Indicates change with *p* < 0.05 compared to control (CON) group.

**Figure 7:**
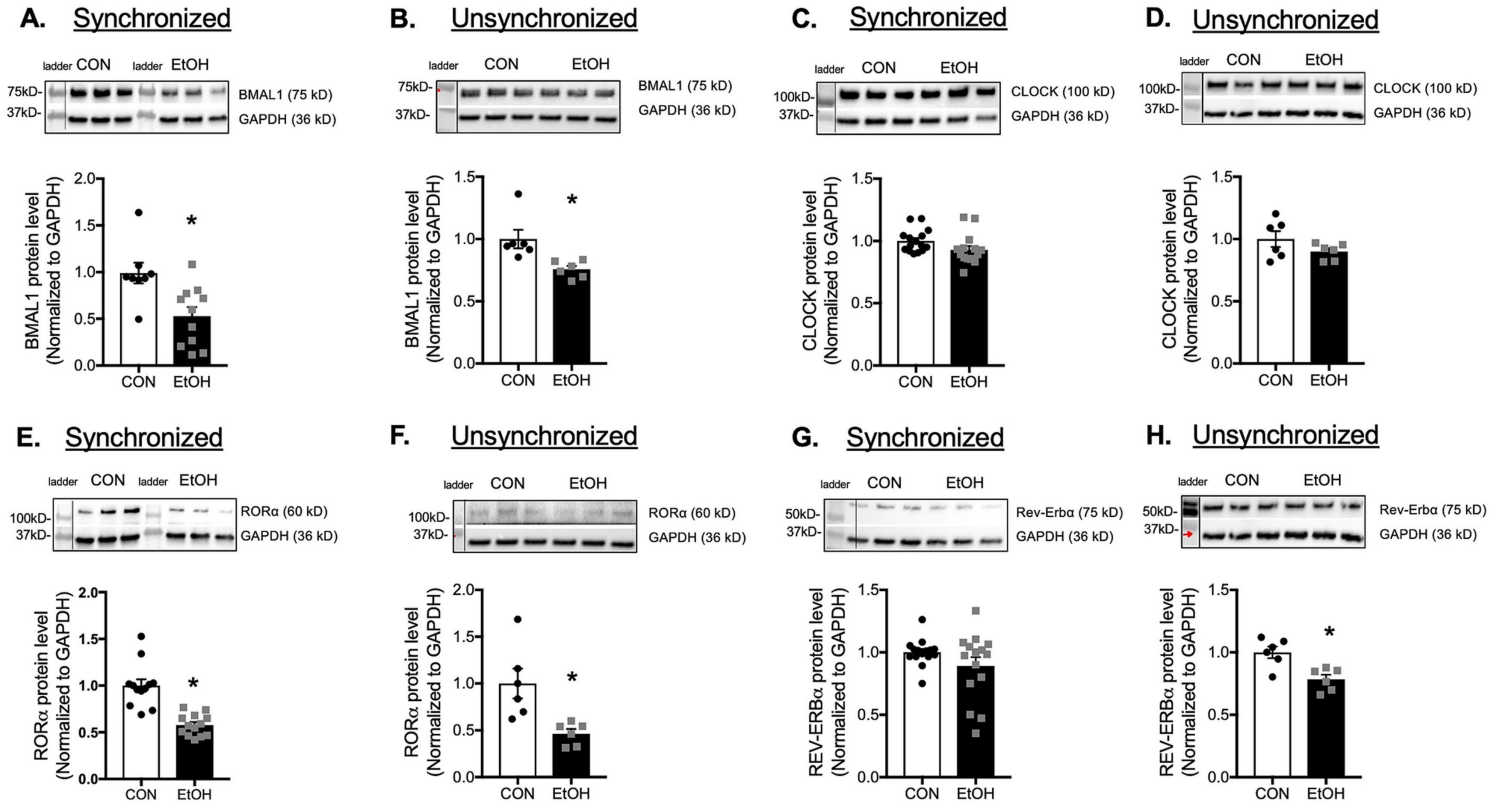
Ethanol treatment inhibited core circadian signaling molecules in murine lung fibroblasts. Protein levels from synchronized and unsynchronized murine primary lung (mPLFs) ± ethanol (EtOH, 60 mM; 72 h) were analyzed for: **(A,B)** BMAL1 (75 kD), **(C,D)** CLOCK (100 kD), **(E,F)** RORα (60 kD), and **(G,H)** REV-ERBα (75 kD) were normalized to GAPDH (36 kD) levels from the same blot and reported as fold-change compared to the untreated (CON) group. Ethanol treatment significantly decreased the BAML1 and RORα protein levels in both synchronized and unsynchronized conditions, decreased REV-ERBα in the unsynchronized condition while it did not change the CLOCK protein expression in either condition. Representative western immunoblots are shown above the graphs. *N* = 8–15 per group. Data are presented as mean ± SE. * Indicates change with *p* < 0.05 compared to control (CON) group.

Lung fibroblasts play a central role in tissue repair and remodeling following injury. Based on the alterations observed in whole lung tissue, we hypothesized that ethanol-treated lung fibroblasts would exhibit similar disruptions in circadian gene expression. To test this, we compared synchronized and unsynchronized primary lung fibroblasts (PLFs) treated with ethanol for 24 h and assessed expression of core circadian genes. Ethanol significantly suppressed *Bmal1* and *Rorα* in both synchronized and unsynchronized conditions (Figures 6A,B,E,F). *Rev-erbα* expression was reduced in synchronized cells but paradoxically increased in unsynchronized cells (Figures 6G,H). In contrast, *Clock* expression was significantly elevated only in synchronized fibroblasts, with no change in the unsynchronized condition (Figures 6C,D). Analysis of downstream BMAL1–CLOCK target genes in synchronized cells revealed marked reductions in *Per1* and *Per2*, while *Cry1*, *Cry2*, and *Rev-erbβ* remained unchanged (Supplementary Figures S1A–E). Protein analyses corroborated these findings: ethanol exposure decreased BMAL1 and RORα in both synchronized and unsynchronized fibroblasts (Figures 7A,B,E,F), and reduced PER1 and PER2 in synchronized cells (Supplementary Figure S2). Notably, CLOCK protein levels were unchanged in both conditions (Figures 7C,D), and REV-ERBα protein was stable in synchronized cells but suppressed in unsynchronized cells (Figures 7G,H). Together, these findings indicate that ethanol disrupts circadian signaling in lung fibroblasts primarily through suppression of BMAL1 and its associated transcriptional network, leading to downstream reductions in PER1, PER2, and RORα.

### Modulation of RORα activity alters TGFβ and α-SMA expression in murine lung fibroblasts

**Figure 8:**
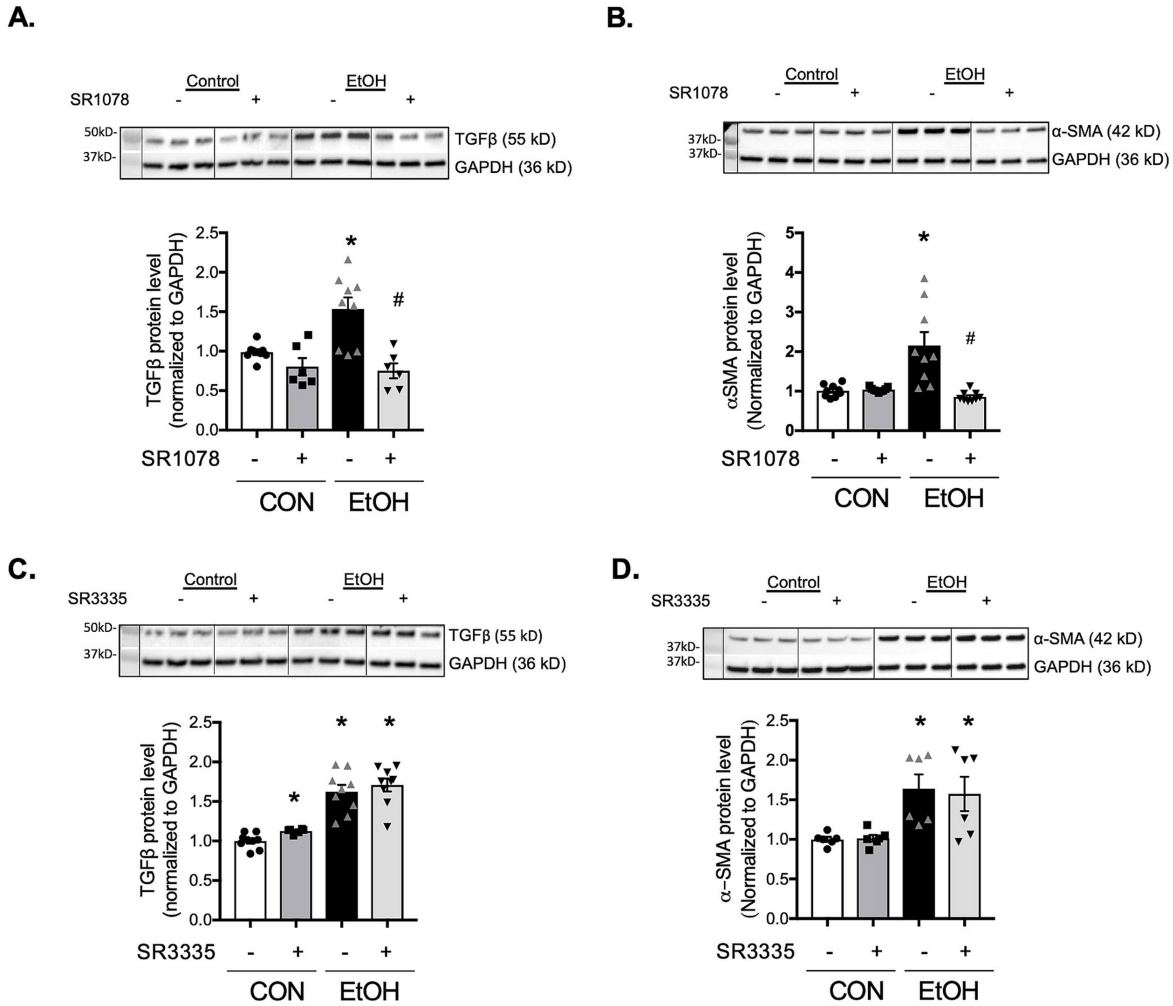
Modulation of RORα activities altered TGFβ and α-SMA expressions in murine lung fibroblasts. Protein levels from murine primary lung fibroblasts (mPLFs) pretreated with the RORα agonist SR1078 (5 μM) or DMSO as a vehicle for 30 min, then ± ethanol (60 mM; 72 h) were analyzed for TGFβ (55 kD) and α-SMA (42 kD). Protein expression was normalized to GAPDH (36 kD) within the same blot and expressed as fold-change relative to the control (CON, vehicle only) group. SR1078 significantly inhibited ethanol-induced **(A)** TGFβ and **(B)** α-SMA protein expression. SR3335 alone significantly upregulated **(C)** TGFβ protein expression while it alone did not alter **(D)** α-SMA protein level. Representative Western blots are shown above the corresponding graphs. *N* = 6–9 per group. Data represents mean ± SE from two independent experiments. * Indicates change with *p* < 0.001 compared to control (CON, vehicle only) group; ^#^ indicates change with *p* = 0.007 compared to ethanol (EtOH) with DMSO treatment.

**Figure 9:**
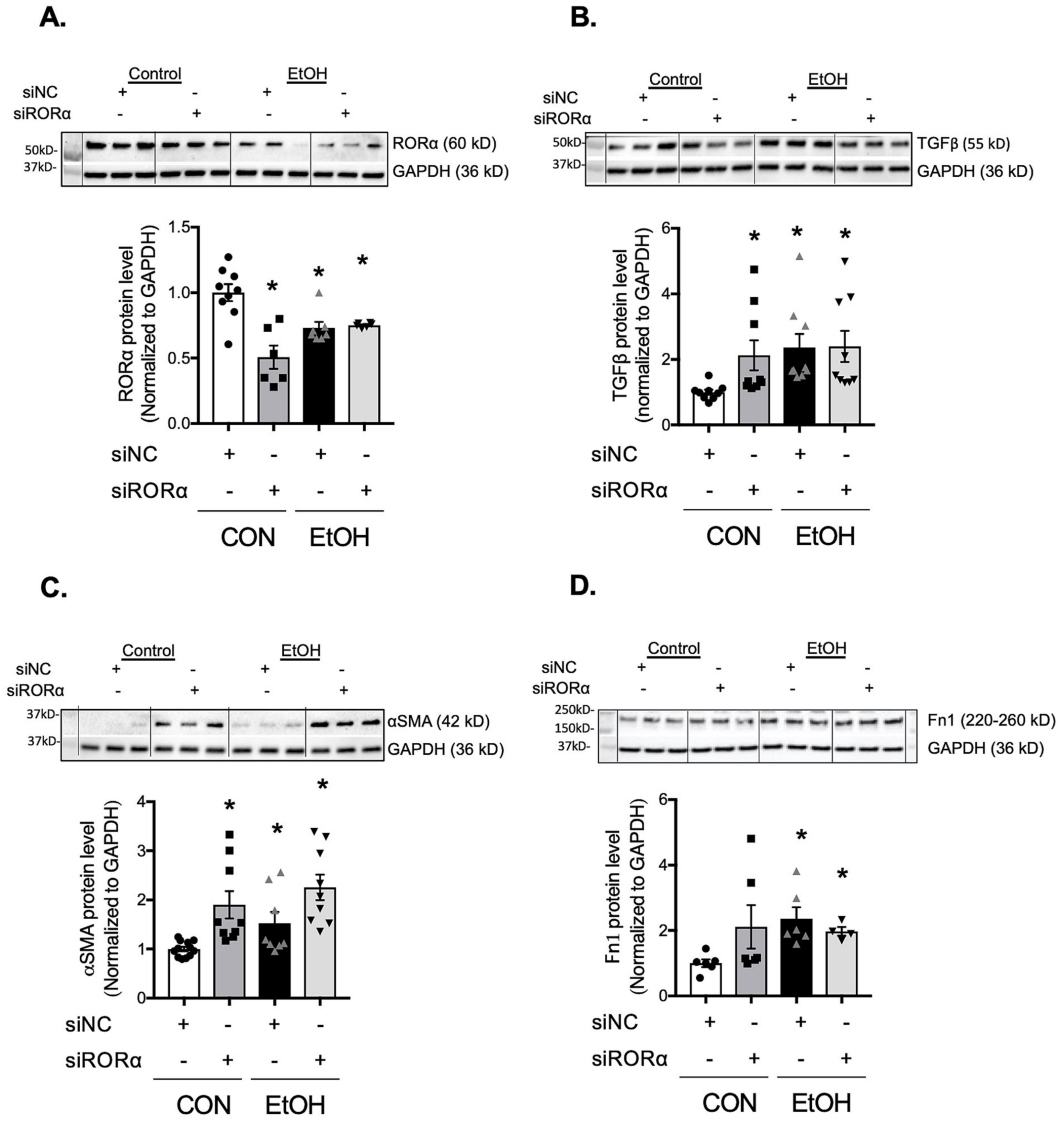
Inhibition of RORα with silencing RNA promoted TGFβ, α-SMA, and fibronectin expressions in murine lung fibroblasts. Protein levels from murine primary lung fibroblasts (mPLFs) transfected with stealth RNAi siRNA targeting RORα (siRORα, 5 nM) or appropriate negative siRNA control (siNC, 5 nM) ± ethanol (60 mM; 48 h) were analyzed for: **(A)** RORα (~60 kD), **(B)** TGFβ (55 kD), **(C)** α-SMA (42 kD), and **(D)** fibronectin (220–260 kD), and normalized with GAPDH (36 kD) levels in the same blot by western analysis. Downregulation of RORα alone significantly increased TGFβ, α-SMA, and a trend to enhance fibronectin protein levels as similar as seen in cells with the ethanol treatment. All data reported as fold-change compared to negative siRNA without ethanol (CON) group. Representative western immunoblots are shown above the corresponding graphs. *N* = 4–12 per group. Data are presented as mean ± SE. * Indicates change with *p* < 0.005 compared with CON group.

RORα has been reported to accelerate BMAL1 transcription and suppress α-SMA expression (33). To examine whether RORα influences ethanol-induced profibrotic markers; TGFβ, α-SMA, and fibronectin, we used both pharmacological and genetic approaches. Activation of RORα with the agonist SR1078 markedly reduced ethanol-induced TGFβ and α-SMA expression to levels comparable to vehicle-treated cells (Figures 8A,B). Conversely, inhibition of RORα with the inverse agonist SR3335 significantly increased TGFβ expression, although not to the same extent as ethanol, and did not alter α-SMA protein levels (Figures 8C,D). Previous work (33) has shown that SR1078 activates transcription driven by ROR target gene promoters in a RORE-dependent manner while inhibiting the constitutive transactivation activity of RORα. Consistent with this, BMAL1 gene expression was elevated in PLFs treated with SR1078 compared to control or ethanol-treated cells (Supplementary Figure S3A). In contrast, SR3335 treatment showed a trend toward decreased BMAL1 expression and did not further reduce BMAL1 when combined with ethanol (Supplementary Figure S3B). To address the potential off-target effects of pharmacological RORα modulators, we additionally silenced RORα using siRNA. Knockdown of RORα decreased BMAL1 protein expression similarly to ethanol treatment, without an additive effect (Supplementary Figure S3C). In parallel, RORα silencing (Figure 9A) reproduced the profibrotic response observed with ethanol, significantly increasing TGFβ and α-SMA (Figures 9B,C), but not fibronectin (Figure 9D), compared to control siRNA-treated cells. Collectively, these findings suggest that RORα plays a partial role in modulating ethanol-induced profibrotic signaling, partially through regulation of BMAL1 and associated pathways.

## Discussion

In this study, we demonstrate that chronic ethanol ingestion disrupts circadian rhythmicity in the lung and alters the temporal regulation of profibrotic markers, including TGFβ, α-SMA, and fibronectin. Ethanol consistently suppresses both the transcription and translation of BMAL1 (aryl hydrocarbon receptor nuclear translocator-like 1, ARNTL1), a core circadian transcription factor, as well as downstream components of the BMAL1-CLOCK network such as PER1, PER2, and RORα in lung fibroblasts. Given that RORα functions as a nuclear receptor that reinforces BMAL1 expression through a positive transcriptional feedback loop, we further examined its contribution to ethanol-induced profibrotic signaling. Pharmacologic inhibition of RORα increased TGFβ expression without affecting α-SMA or fibronectin, whereas RORα silencing elevated both TGFβ and α-SMA. Conversely, activation of RORα with the agonist SR1078 attenuated ethanol-induced increases in TGFβ and α-SMA. Together, these findings identify RORα as a key regulator of ethanol-driven profibrotic responses and provide, to our knowledge, the first evidence that chronic ethanol exposure disrupts circadian signaling pathways within the lung.

Chronic ethanol ingestion is a known risk factor for developing acute respiratory distress syndrome (ARDS) in clinical settings (10). In our experimental model, we demonstrated that ethanol promotes fibroblast-to-myofibroblast differentiation (FMD), contributing to fibroproliferative maladaptive repair through exaggerated TGFβ expression in the lung (24). Additionally, we showed that oxidative stress is an early event following ethanol exposure, initiating a feedforward loop that amplifies TGFβ induction and activation (34). Both ethanol and oxidative stress are known disruptors of circadian signaling in the brain and peripheral tissues (7). Lastly, Finger et al. (35) reported that TGFβ plays a critical role in maintaining peripheral clock synchronization among cells. Accordingly, we speculated that ethanol exposure may disrupt circadian signaling in the lung. Our data confirmed that chronic ethanol ingestion disrupts lung circadian oscillation as shown by a shift in lung’s PER2 luciferase activity in comparison to control lungs. However, the precise mechanism by which ethanol disrupts lung circadian signaling is unknown.

More than half of protein-coding genes exhibit circadian oscillation, with distinct patterns across various tissues (7). As discussed above, TGFβ and circadian signaling share a complex, bidirectional relationship (35). In this study, *Tgfβ* transcription exhibited a clear diurnal oscillation in control animals; however, this rhythmicity was absent in the lungs of ethanol-fed animals, which instead showed persistently elevated *Tgfβ* levels throughout the 24-h cycle. In contrast to *Tgfβ*, ethanol induced rhythmic expression of representative profibrotic markers; specifically, *α-sma*, and *fibronectin*, whereas control lungs displayed non-rhythmic and consistently suppressed expression of these genes. Further, there is also a shift in protein peaks from their corresponding mRNA rhythm supporting the idea that circadian dysregulation contributes to aberrant extracellular matrix remodeling. Whether a regulatory factor in control animals inhibits TGFβ-driven transcription of α-SMA, and fibronectin, or whether persistently elevated TGFβ in ethanol-fed animals drives altered activation of these profibrotic markers remains to be determined. These findings suggest that TGFβ oscillation plays a critical role in maintaining tissue homeostasis under normal conditions, and that disruption of this rhythmic pattern or sustained elevation of TGFβ may contribute to maladaptive tissue repair. We speculate that persistent elevation of TGFβ in ethanol-fed animals may override normal inhibitory mechanisms, promoting fibrosis through altered circadian regulation.

The circadian pathway operates through transcriptional-translational feedback loops (7). The CLOCK-BMAL1 complex activates PER and CRY, whose dimers inhibit CLOCK-BMAL1, forming a negative loop (36). ROR (α, β, γ) and REV-ERB (α, β) modulate BMAL1 by competing for ROR elements; ROR promotes, whereas REV-ERB represses its expression (36). Our next question was: how does ethanol disrupt lung circadian rhythm? Ethanol consumption has been shown to alter the expression of core circadian genes, including BMAL1, Cry1/2, and Per1/2, in immune cells, liver, and skeletal muscle, leading to desynchronization between central and peripheral clocks (37–39). Consistent with these reports, we found that chronic ethanol ingestion alters both the expression and oscillatory patterns of core circadian genes, including *Bmal1*, *Clock*, *Rorα*, and *Rev-erbα*. These findings provide a dynamic view of circadian signaling molecules in the lungs of ethanol-fed animals. To fully characterize how these transcriptional changes translate into functional alterations of the circadian clock, we next examined whether ethanol-induced disruptions in gene expression were reflected at the protein level. In the present study, chronic ethanol ingestion disrupted both the transcriptional and translational rhythms of key circadian signaling regulators, including BMAL1, CLOCK, RORα, and REV-ERBα. Notably, the frequent misalignment between mRNA and protein abundance; such as BMAL1 suppression at the transcript level during early and late phases but reduced protein only at ZT12, or the broad upregulation of CLOCK mRNA contrasted with a protein increase restricted to ZT0, suggests that ethanol alters not only gene expression but also post-transcriptional and post-translational regulatory mechanisms. Together, these findings support a model in which ethanol-induced circadian disruption contributes to maladaptive tissue remodeling and may predispose the lung to enhanced fibrotic susceptibility.

Because the lung comprises multiple cell types; and given the critical role of fibroblasts in tissue repair, along with our previous evidence that ethanol promotes a pro-fibrotic phenotype in these cells, we focused our analysis on lung fibroblasts (11, 34). We first compared the synchronized condition with an unsynchronized condition to assess whether the effect of ethanol is intrinsic or dependent on external timing cues. We found that ethanol suppresses BMAL1 gene and protein expression in both synchronized and unsynchronized conditions, while upregulating CLOCK gene transcription only in synchronized cells, without a corresponding increase in CLOCK protein levels. This pattern suggests that CLOCK upregulation may represent a compensatory response to disruption of the core circadian machinery, with BMAL1 serving as a primary and more ethanol-sensitive target. Ethanol also produced opposing effects on Rev-erbα expression; downregulating it in synchronized cells but upregulating it in unsynchronized cells, suggesting that REV-ERBα regulation is highly phase-dependent and that ethanol interacts differently with the clock network depending on cellular circadian alignment. Together, these findings indicate that while BMAL1 and RORα are consistently suppressed regardless of synchronization state, CLOCK and REV-ERBα exhibit phase-specific or low-amplitude rhythms that make their responses detectable only under synchronized conditions, highlighting differential sensitivity of circadian components to ethanol. The discrepancy between mRNA and protein levels of CLOCK and *REV-ERBα* may result from post-transcriptional regulatory mechanisms, such as epigenetic mechanism (i.e., micro-RNA) or environmental influences (e.g., high-fat diet, LPS, UV exposure). Specifically, ethanol has been shown to induce epigenetic changes, and future studies aimed at further dissecting this regulatory mechanism are warranted. Although our current work focuses on the role of RORα, it is important to note that REV-ERBα may also play a critical role in this model. Previous studies have demonstrated its involvement in pulmonary fibrosis pathogenesis: mutations in *Rev-erbα* exacerbate bleomycin-induced fibrosis whereas activation of REV-ERBα inhibits TGFβ-induced fibroblast-to-myofibroblast transition (FMT) and reduces extracellular matrix (ECM) production. Accordingly, future studies assessing the impact of RORα and REV-ERBα imbalance on ethanol-mediated BMAL1 may provide deeper insight into the mechanisms driving ethanol-induced alterations in lung molecular phenotype.

Our study provides new evidence that chronic ethanol ingestion disrupts lung circadian signaling, which may contribute to maladaptive tissue repair. Chronic ethanol exposure has previously been linked to fibroproliferative repair following acute lung injury in murine models, mediated by ethanol-induced fibroblast-to-myofibroblast differentiation (9, 22). In this study, we found that chronic alcohol ingestion significantly suppressed the expression of Bmal1 and Rorα. BMAL1, a core circadian clock component, regulates numerous downstream genes, so its modulation can broadly influence multiple physiological systems. RORα, a transcriptional activator of BMAL1, also impacts development, metabolism, and inflammation. However, as a downstream regulator of BMAL1, RORα represents a more specific and potentially therapeutic target; therefore, we prioritized investigating RORα in our experimental model. Our data reveal an inverse relationship between RORα activation and the expression of TGFβ and α-SMA under ethanol stimulation, suggesting that RORα may play a protective role against ethanol-induced profibrotic signaling. Notably, activation of RORα with SR1078 significantly suppressed TGFβ and α-SMA, whereas inhibition of RORα using an inverse agonist or siRNA did not fully replicate the profibrotic effects of ethanol. This discrepancy may indicate that ethanol-induced circadian disruption involves multiple regulatory molecules beyond RORα, including non-coding RNAs, which collectively modulate fibroblast activation. Additionally, ethanol may exert a dominant suppressive effect on BMAL1 and its downstream targets, creating a broader transcriptional imbalance that cannot be mimicked by RORα inhibition alone. Although ethanol-driven suppression of BMAL1 appears to be an early event leading to downstream effects, RORα activation can attenuate ethanol-induced profibrotic phenotypes in lung fibroblasts. Future studies are warranted to elucidate the mechanisms underlying ethanol-mediated circadian disruption. Overall, these findings underscore the complexity of circadian regulation in lung fibroblasts and suggest that targeting RORα activation, rather than inhibition, may offer therapeutic potential for mitigating ethanol-induced fibrotic remodeling.

This study has several limitations. First, we examined only a single ethanol concentration to maintain consistency with previous reports; future studies should evaluate a range of doses *in vivo* and *ex vivo* to better define dose-dependent effects. Second, we analyzed a limited set of circadian and profibrotic molecules; expanding this panel will provide a more comprehensive view of ethanol-induced signaling changes in lung cells. Third, the causal relationship between BMAL1 and RORα remains unclear. Whether ethanol-induced alterations in BMAL1 drive RORα downregulation or vice versa is unknown. Either molecule could initiate a cycle of negative regulation, creating a feedback loop. Because BMAL1 is upstream of multiple transcription factors, its manipulation may have broad off-target effects; therefore, we focused on RORα as a more specific downstream target. Fourth, our *in vitro* studies did not include comparisons between synchronized and unsynchronized conditions with temporal sampling across the 24-h cycle, limiting our ability to fully characterize dynamic circadian responses. Future studies will incorporate higher-resolution time-series analyses to capture these oscillatory patterns. Finally, based on the in vitro data presented here, future in vivo studies using RORα agonists or RNA silencing will be essential to confirm the effects of pharmacological modulation and determine its role in disease-relevant models, thereby assessing translational potential.

In conclusion, our findings provide new evidence that chronic ethanol ingestion disrupts lung circadian signaling, likely through initial suppression of BMAL1, which in turn downregulates downstream genes such as RORα. This disruption of circadian signaling appears to contribute to altered rhythmicity of profibrotic markers (TGFβ, α-SMA, Fn1), leading to maladaptive tissue repair. Importantly, activation of RORα mitigates ethanol-induced expression of profibrotic markers, including TGFβ and α-SMA, in primary lung fibroblasts. These results suggest a potential avenue for developing preventive or therapeutic strategies to enhance tissue repair and recovery following injury in this at-risk population.

## Data Availability

The raw data supporting the conclusions of this article will be made available by the authors, without undue reservation.

## Footnotes

1
https://nitecap.org
