# Supplementary material for: Downregulation of RORα by alcohol promotes TGFβ and α-SMA expression in mouse lung fibroblasts
Source: Front Med (Lausanne). 2026 Feb 4;13:1719787. doi: 10.3389/fmed.2026.1719787 (PMC12913583; doi:10.3389/fmed.2026.1719787)
Supplement: Supplementary file 1 [file Data_Sheet_1.pdf]

Supplementary Data: Down-regulation of RORα by alcohol promotes TGFβ and α-SMA expression in mouse lung fibroblasts

Running Title: Ethanol **disrupts lung and fibroblast circadian signaling**

Xian Fan[1], Hui Tao[1], Bum-Yong Kang[2], Nicolas Diaz[3], Kenkichi Baba[3], GianlucaTosini[3], Justin Guo[1], Samantha M. Yeligar[1,4], and Viranuj Sueblinvong[1*]

[1] Emory University School of Medicine, Department of Medicine, Division of Pulmonary, Allergy, Critical Care, and Sleep Medicine, Atlanta, GA 30322

[2] Emory University School of Medicine, Department of Pediatrics, Division of Pulmonary, Allergy, Sleep, and Cystic Fibrosis, Atlanta, GA 30322

[3] Morehouse School of Medicine, Department of Pharmacology and Toxicology, Atlanta GA 30310

[4] Atlanta Veterans Affairs Health Care System, Decatur, GA 30033

***Correspondence to**: Viranuj Sueblinvong (ORCID ID 0000-0002-4770-5900)

615 Michael Street, NE, Suite 205, Atlanta, GA 30322

email: vsuebli@emory.edu

Tel: 404-727-9560

Fax : 404-712-2974

ORICD: 0000-0002-4770-5900

Supplementary Table

Table S1. The effect of chronic ethanol ingestion on the circadian signaling molecules and fibroblast-to-myofibroblast markers gene rhythmicity analyzed by using JTK_CYCLE.

**Gene**	**CON**	**EtOH**
bmal1	Yes	Yes
clock	Yes	Yes
rorα	Yes	Yes
rev-ernα	Yes	Yes
tgfβ	Yes	No
α-sma	No	Yes
fibronectin	No	Yes

Supplementary Figures

Figure S1. Ethanol exposure *in vitro* inhibited key molecules in the circadian signaling pathway. Gene expression levels from murine primary lung fibroblasts (mPLFs) ± ethanol (EtOH, 60 mM; 24 hours) were analyzed for: (**A**) *Cry1*, (**B**) *Cry2*, (**C**) *Per1*, (**D**) *Per2,* and (**E**) *Rev-erbβ* were analyzed for mRNA expression by real-time qPCR. Ethanol exposure significantly suppressed *Per 1* and *Per2* gene expression but didn't affect other circadian gene expressions. Data are presented as mean ± SE from two separate experiments. N = 4-6. Data are presented as mean ± SE. *Indicates change with $p < 0.05$ compared to control (CON) group.

Figure S2. Ethanol treatment inhibited core circadian signaling molecules in murine lung fibroblasts. Protein levels from murine primary lung fibroblasts (mPLFs) ± ethanol (EtOH, 60 mM; 72 hours) were analyzed for: (**A**) PER1 (~80 kD, N = 4/group) and (**B**) PER2 (~120 kD, N = 10/group) by Western Immunoblot. The targeting protein levels were normalized to GAPDH (36 kD) levels from the same blot and reported as fold-change compared to the untreated (CON) group. Both PER1 and PER2 protein expressions were significantly inhibited. Representative Western Immunoblots are shown above the graphs. Data are presented as mean ± SE. *Indicates change with p<0.05 compared to control (CON) group.

Figure S3. Modulation of RORα with RORα agonist or silencing RNA activated or suppressed BMAL1 in murine lung fibroblasts. Murine primary lung fibroblasts (mPLFs) treated with either (**A**) RORa agonist (SR1078, 5 μM) ± ethanol (60 mM, 24 hours) or (**B**) RORa inverse agonist (SR3335, 5 μM) ± ethanol (60 mM, 24 hours) were analyzed for BMAL1 gene expression by qPCR. SR1078 significantly increased BMAL1 gene expressions in the absence or presence of ethanol while SR3335 showed a trend to decrease BMAL1. Cells transfected with (**C**) siRNA targeting RORα (siRORα, 5 nM) or negative siRNA control (siNC, 5 nM) ± ethanol (60 mM, 48 hours) then analyzed for BMAL1(75 kDa) and normalized with GAPDH (36 kD) levels in the same blot by Western analysis. Down-regulation of RORα by silencing RNA significantly decreased BMAL1 protein levels in ± ethanol treatment. All data reported as fold-change compared to untreated control (CON) group. Representative Western Immunoblots are shown above the corresponding graphs. N = 3-6. Data are presented as mean ± SE. * Indicates change with $p<0.005$ compared with CON group and $^{\#}$indicates change with $p<0.05$ compared with EtOH group.

Figure S4. Corresponding Western blot gels for representative data in Figure 3.

Figure S5. Corresponding Western blot gels for representative data in Figure 5.

Figure S6. Corresponding Western blot gels for representative data in Figure 7 and Supplementary Figure S2.

Figure S7. Corresponding Western blot gels for representative data in Figure 8. (**A**)

Figure S8. Corresponding Western blot gels for representative data in Figure 9.

Figure S9. Corresponding Western blot gels for representative data in supplementary Figure S3.
