# Supplementary figures and images for: Downregulation of RORα by alcohol promotes TGFβ and α-SMA expression in mouse lung fibroblasts
Source: Front Med (Lausanne). 2026 Feb 4;13:1719787. doi: 10.3389/fmed.2026.1719787 (PMC12913583; doi:10.3389/fmed.2026.1719787)

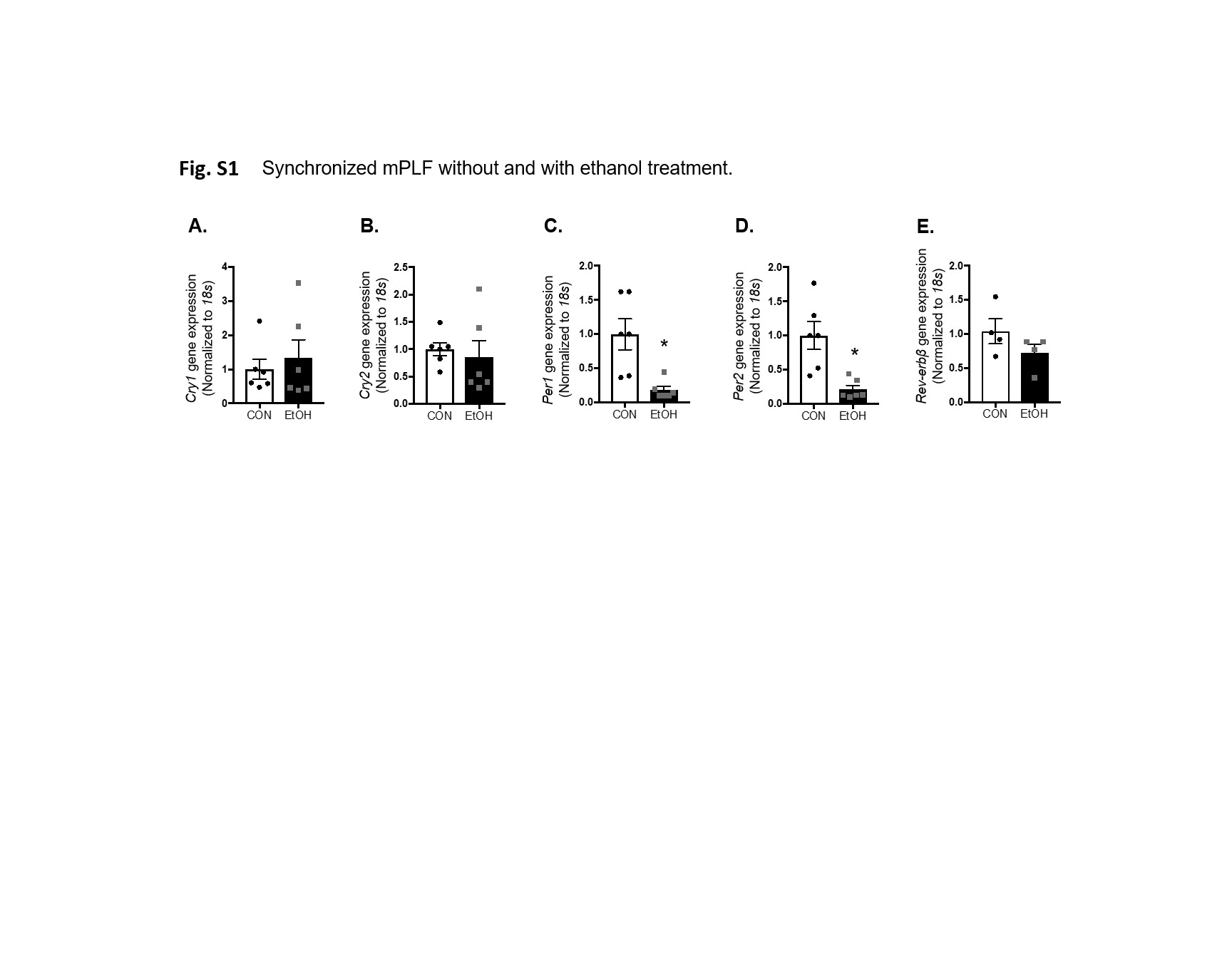

Supplement: Supplementary file 2 [file Image_1.jpeg]

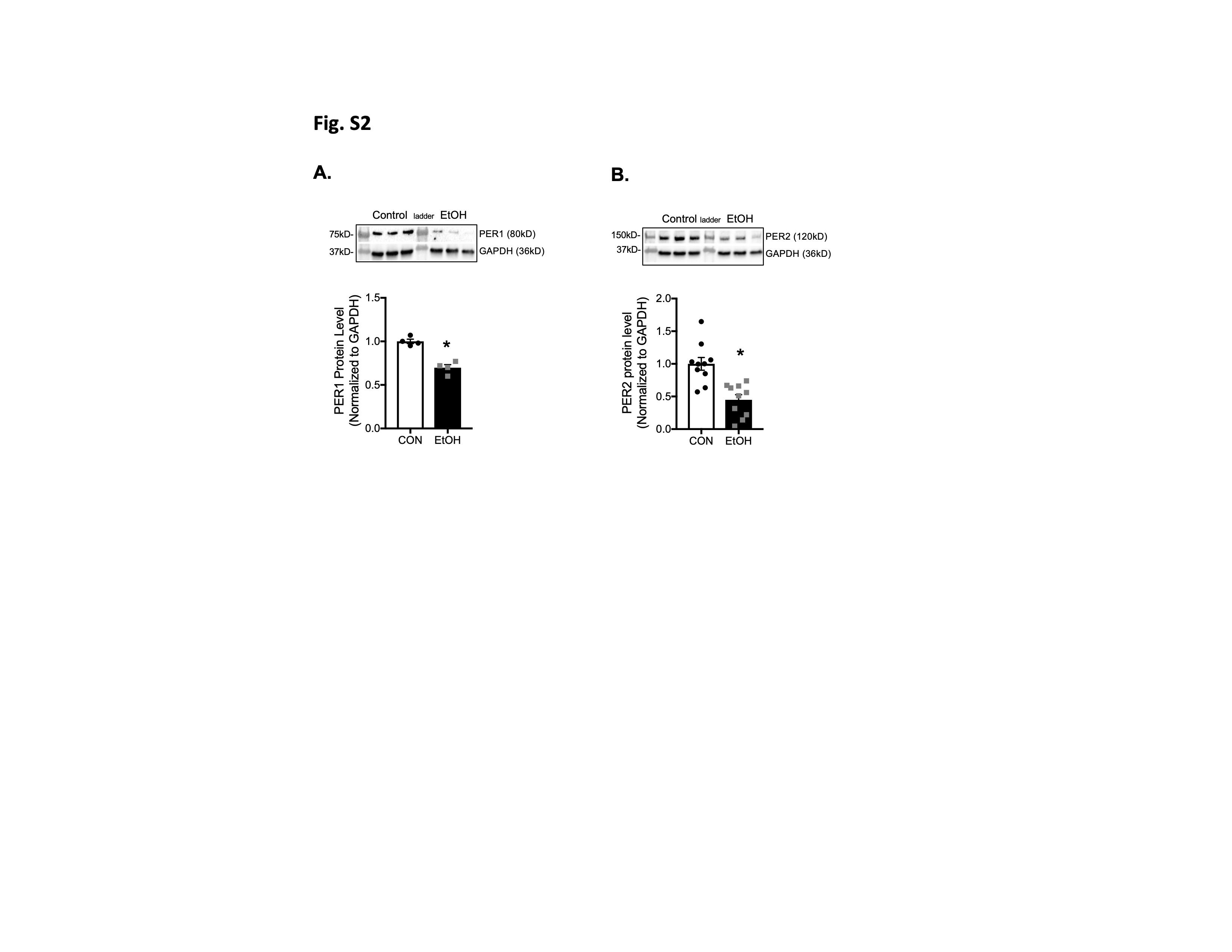

Supplement: Supplementary file 3 [file Image_2.jpeg]

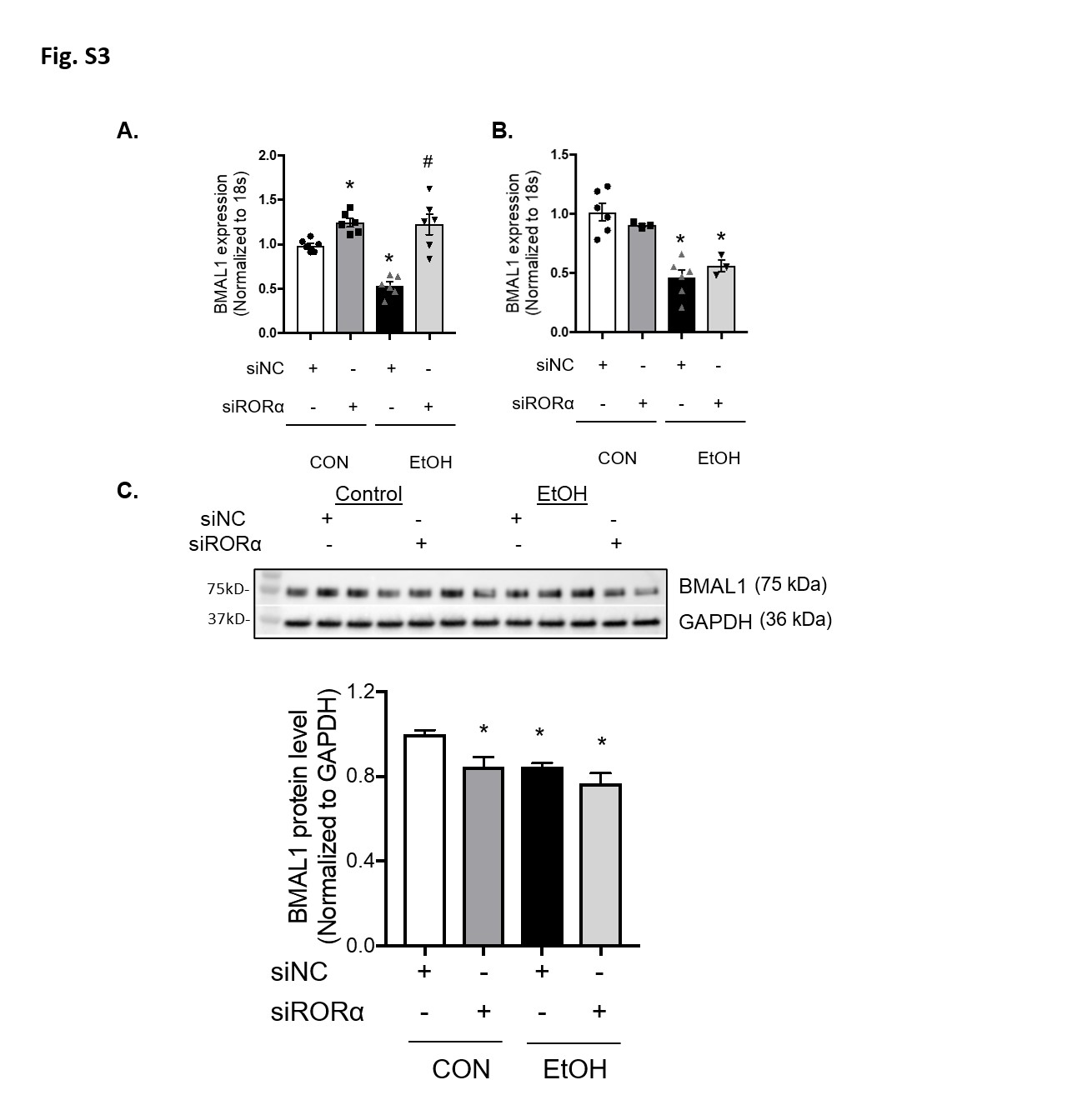

Supplement: Supplementary file 4 [file Image_3.jpeg]

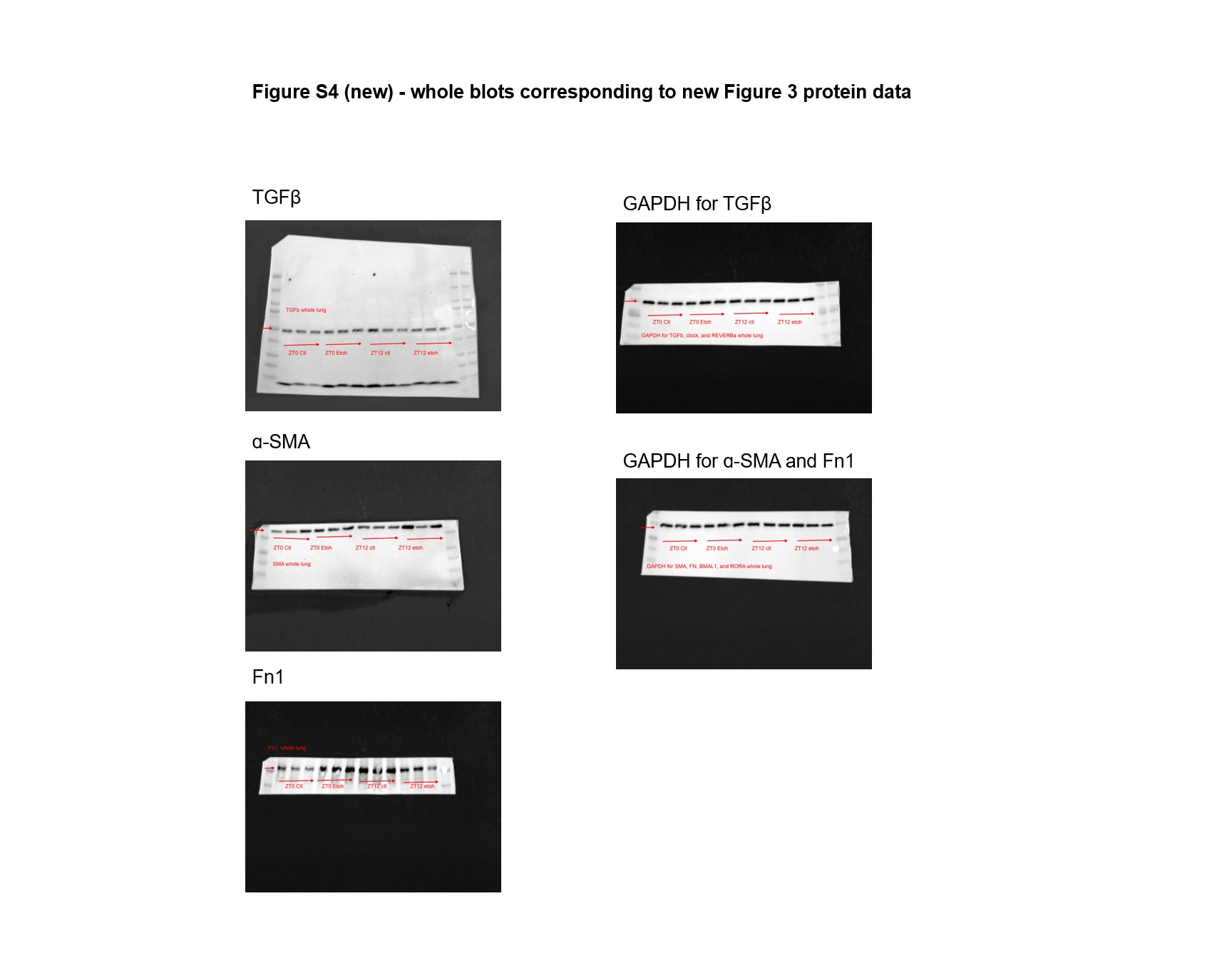

Supplement: Supplementary file 5 [file Image_4.jpeg]

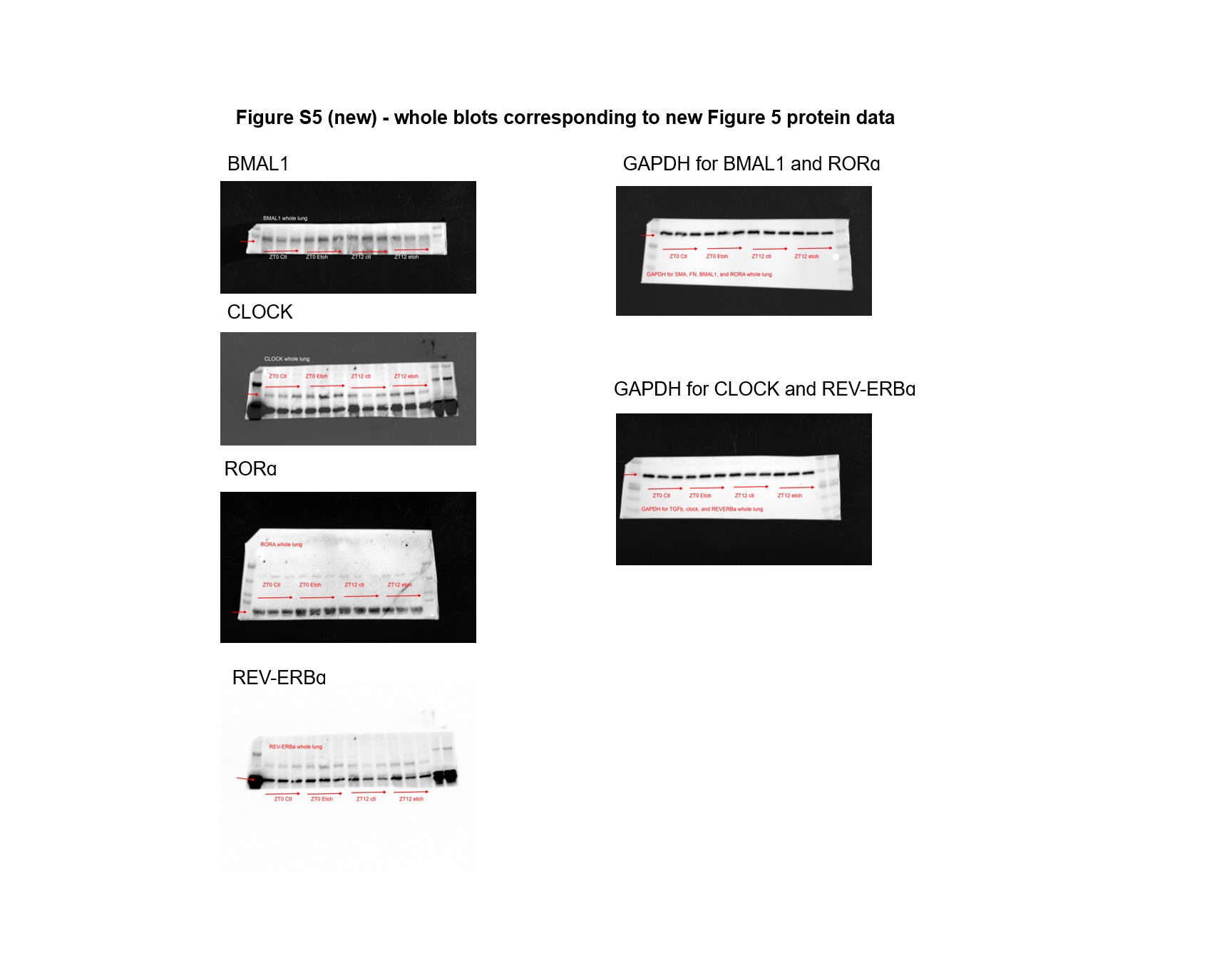

Supplement: Supplementary file 6 [file Image_5.jpeg]

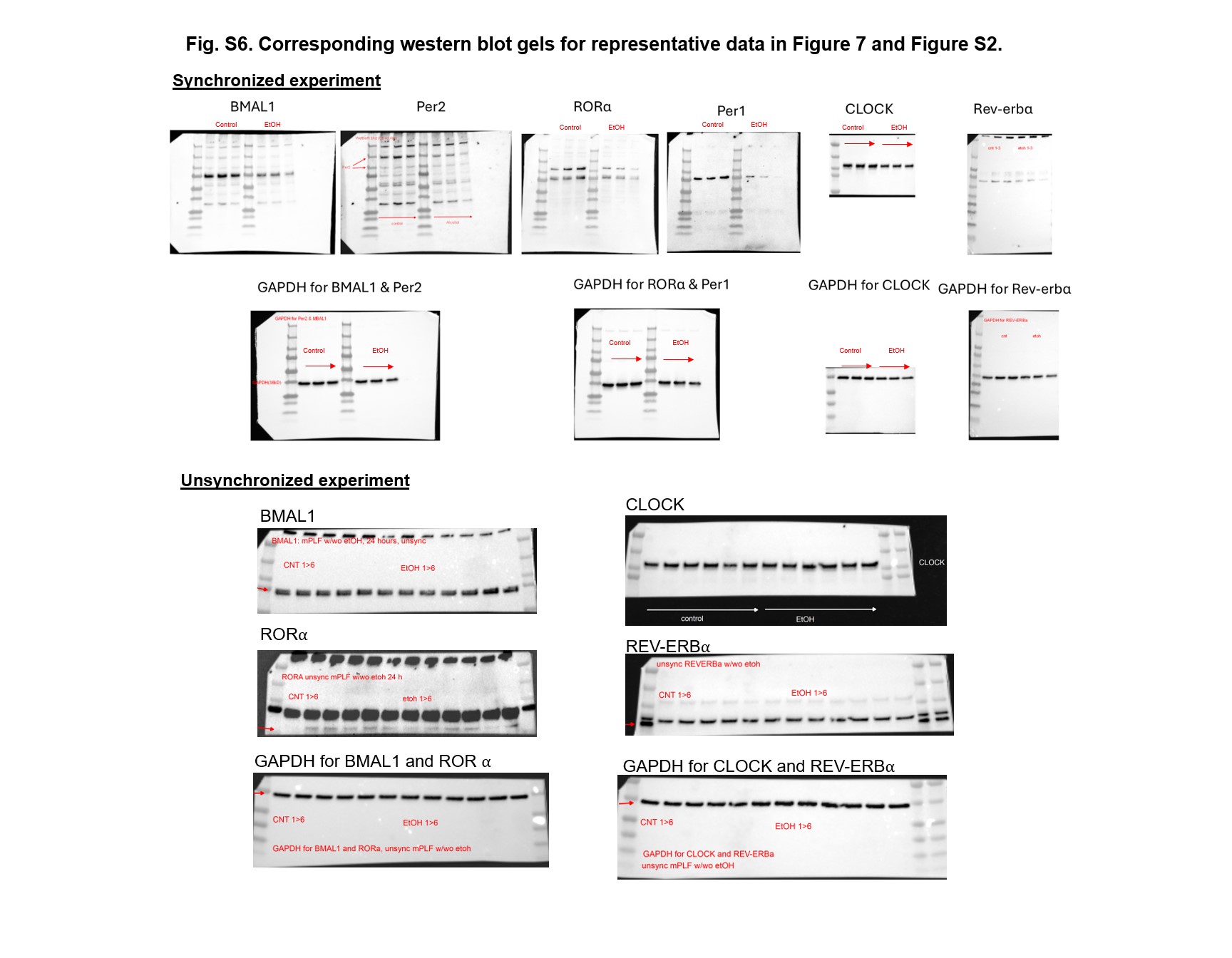

Supplement: Supplementary file 7 [file Image_6.jpeg]

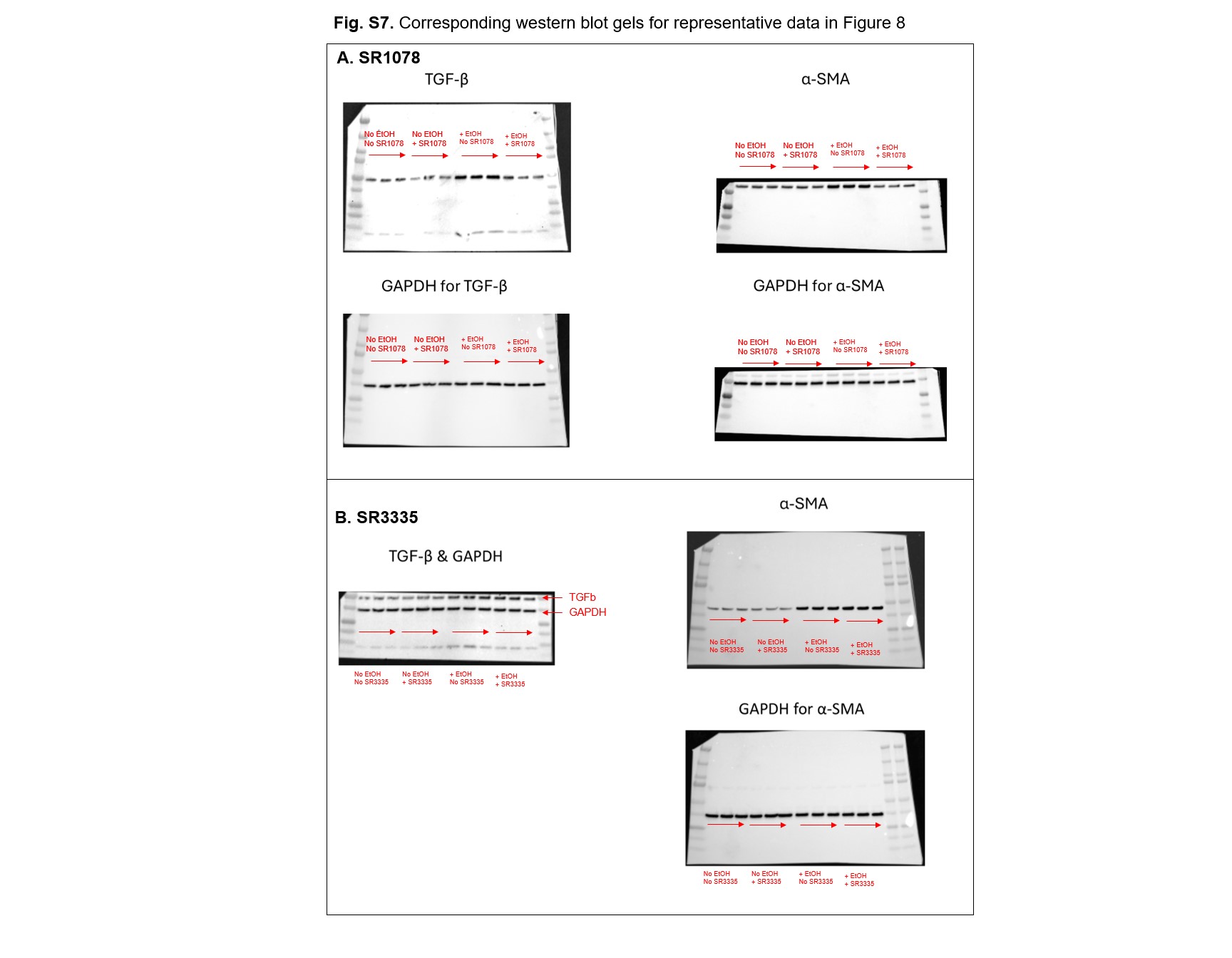

Supplement: Supplementary file 8 [file Image_7.jpeg]

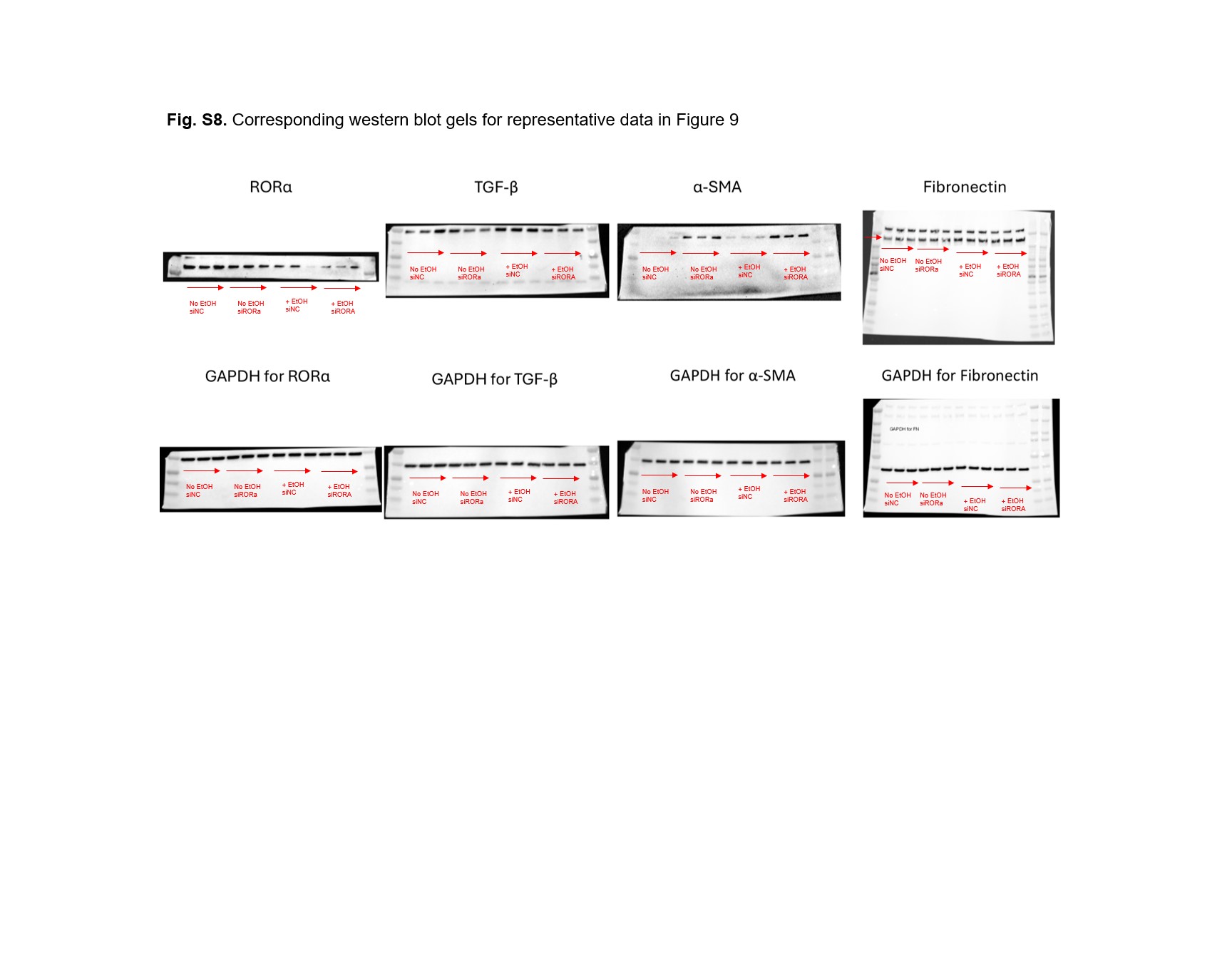

Supplement: Supplementary file 9 [file Image_8.jpeg]

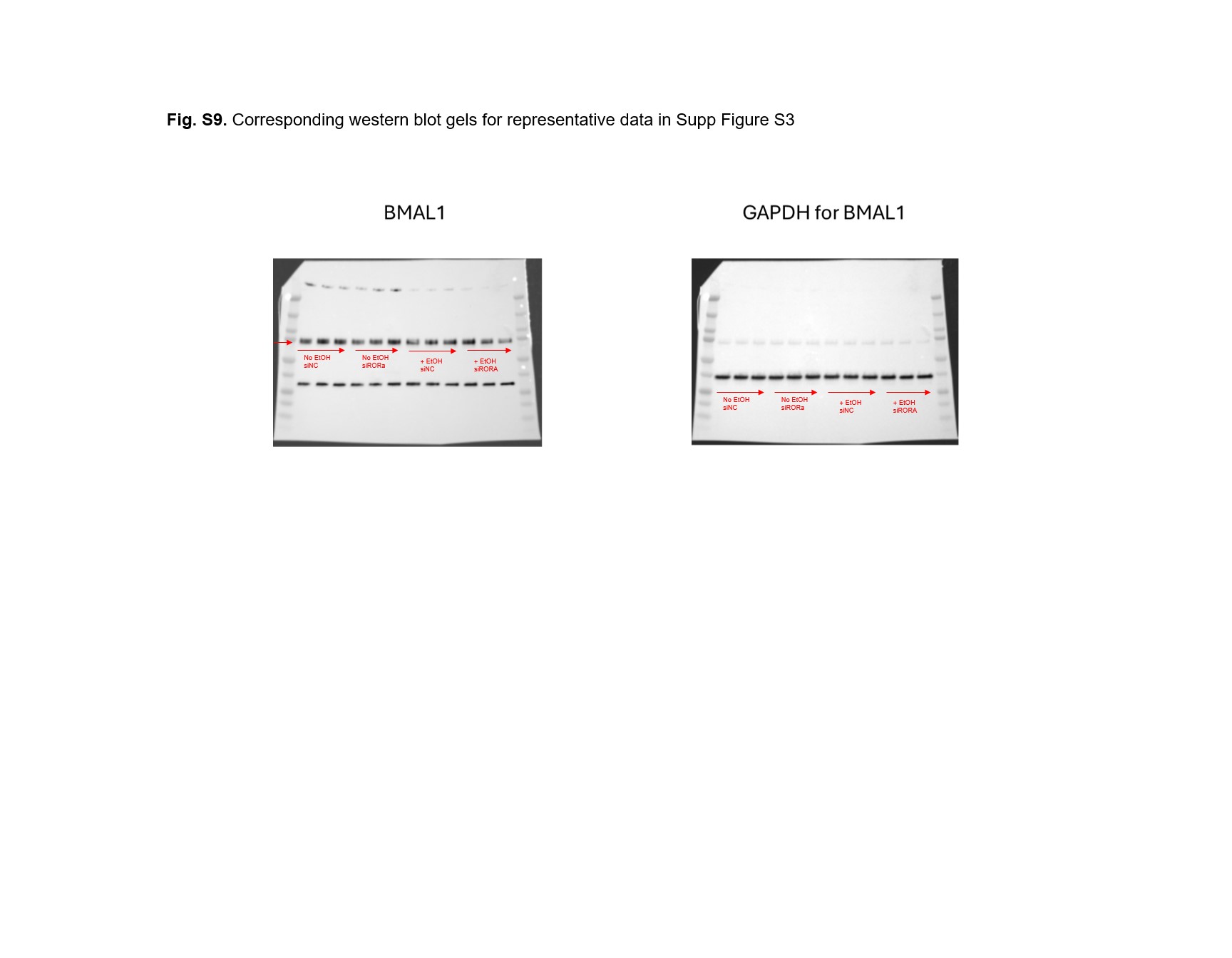

Supplement: Supplementary file 10 [file Image_9.jpeg]
